# Analysis of the cells isolated from epithelial cell rests of Malassez through single-cell limiting dilution

**DOI:** 10.1038/s41598-021-04091-0

**Published:** 2022-01-10

**Authors:** Syed Taufiqul Islam, Yoshihito Kurashige, Erika Minowa, Koki Yoshida, Durga Paudel, Osamu Uehara, Yunosuke Okada, Dembereldorj Bolortsetseg, Sayaka Sakakibara, Yoshihiro Abiko, Masato Saitoh

**Affiliations:** 1 Division of Pediatric Dentistry, Department of Oral Growth and Development, School of Dentistry, Health Sciences University of Hokkaido, 1757 Kanazawa, Ishikari-Tobetsu, Hokkaido, 061-0293 Japan; 2 Division of Oral Medicine and Pathology, Department of Human Biology and Pathophysiology, School of Dentistry, Health Sciences University of Hokkaido, 1757 Kanazawa, Ishikari-Tobetsu, Hokkaido, 061-0293 Japan; 3 Advanced Research Promotion Center, Health Sciences University of Hokkaido, 1757 Kanazawa, Ishikari-Tobetsu, Hokkaido, 061-0293 Japan; 4 Division of Disease Control and Molecular Epidemiology, Department of Oral Growth and Development, School of Dentistry, Health Sciences University of Hokkaido, 1757 Kanazawa, Ishikari-Tobetsu, Hokkaido, 061-0293 Japan

**Keywords:** Biochemistry, Cell biology, Molecular biology

## Abstract

The epithelial cell rests of Malassez (ERM) are essential in preventing ankylosis between the alveolar bone and the tooth (dentoalveolar ankylosis). Despite extensive research, the mechanism by which ERM cells suppress ankylosis remains uncertain; perhaps its varied population is to reason. Therefore, in this study, eighteen unique clones of ERM (CRUDE) were isolated using the single-cell limiting dilution and designated as ERM 1–18. qRT-PCR, ELISA, and western blot analyses revealed that ERM-2 and -3 had the highest and lowest amelogenin expression, respectively. Mineralization of human periodontal ligament fibroblasts (HPDLF) was reduced in vitro co-culture with CRUDE ERM, ERM-2, and -3 cells, but recovered when an anti-amelogenin antibody was introduced. Transplanted rat molars grown in ERM-2 cell supernatants produced substantially less bone than those cultured in other cell supernatants; inhibition was rescued when an anti-amelogenin antibody was added to the supernatants. Anti-Osterix antibody staining was used to confirm the development of new bones. In addition, next-generation sequencing (NGS) data were analysed to discover genes related to the distinct roles of CRUDE ERM, ERM-2, and ERM-3. According to this study, amelogenin produced by ERM cells helps to prevent dentoalveolar ankylosis and maintain periodontal ligament (PDL) space, depending on their clonal diversity.

## Introduction

The PDL is a highly specialized cellular connective tissue that attaches the cementum to the surrounding alveolar bone and holds the tooth in the alveolar socket^1,2^. Maintaining the space between the cementum and the alveolar bone is one of PDL's fundamental properties. PDL serves as a barrier to protect the root by limiting possible bone ingrowth and eventual root resorption. Therefore, the absence of PDL can lead to early tooth loss and dentoalveolar ankylosis^3^. Early-onset dentoalveolar ankylosis due to periodontal injuries or reimplanted avulsed tooth tends to have a negative impact on occlusion. The most common consequences are infraocclusion and vertical bone defect, tipping of adjacent tooth into the space of infraocclusion resulting in loss of arch shape, supra eruption of opposing tooth, dental asymmetry, midline deviation and impaction of the ankylosed tooth and its successor, and deflected eruption trajectory of successors^4,5^. Dentoalveolar ankylosis also occurs after surgical endodontic treatments, including intentional extraction and repositioning of the tooth^6^. However, it should be emphasized that dentoalveolar ankylosis is not only biologically but also biomechanically related to dental prognosis, as it is inevitably associated with the loss of PDL^7^. ERM, the only odontogenic epithelial cells in the PDL tissue, remain quiescent throughout their lifetime. ERM has been shown to maintain the space, homeostasis, and regeneration of PDL^8^. ERM maintains the PDL space by inhibiting cemento-osteogenesis thereby preventing dentoalveolar ankylosis. Conversely, ERM is also thought to be involved in cementum formation during tooth root development^2,9–12^. Several studies have confirmed the expression of different types of proteins, categorized as cytokeratin’s (ck), bone matrix proteins, and enamel matrix proteins (EMPs), in ERM^13^. EMPs are also the major secretory proteins of ameloblast cells during amelogenesis; they comprise mainly of amelogenin (90%) and non-amelogenin proteins (enamelin, ameloblastin, amelotin, and tuftelin). Amelogenin, the primary protein of EMPs, is one of the most extensively studied proteins in the human body. It plays a significant role in enamel biomineralization during amelogenesis and dictates the width and thickness of the apatite crystals. Amelogenin has been reported to maintain the PDL space^14^ and enhance human cementoblast mineralization^15^. Generally, cells from a subpopulation show harmony in their morphology and function. However, recent reports have indicated that cells from the same cell line have different morphologies, molecular expression, and functions^16,17^. The ERM is composed of various cell populations^18^, which might account for the different functions of this structure. Although a few cell subtypes in the ERM have been identified in a morphological study^18^, the isolation of ERM clones from the different cell populations has not been attempted thus far. We anticipated that the isolation of single-cell clones from the ERM might aid in understanding the various functions of this entity. Therefore, the present study aimed to establish and investigate clone cells derived from the ERM using the single-cell limiting dilution method.

The characteristics of individual ERM clones were determined by evaluating the cell growth rate, secretion of EMPs, and expression levels of the markers of outer enamel epithelial (OEE) and inner enamel epithelial (IEE) cells. The ERM clones were co-cultured with HPDLF cells to observe the effect of each clone cell on HPDLF cell mineralization. The roles of these cells in regulating mineralization and maintaining the PDL spaces were examined in vivo. Additionally, bioinformatics analysis of NGS data were used to identify the genes associated with differential functions among the isolated clones.

## Results

### Isolated clones from epithelial-like cells exhibited different cell morphologies and proliferation ratios

**Figure 1:**
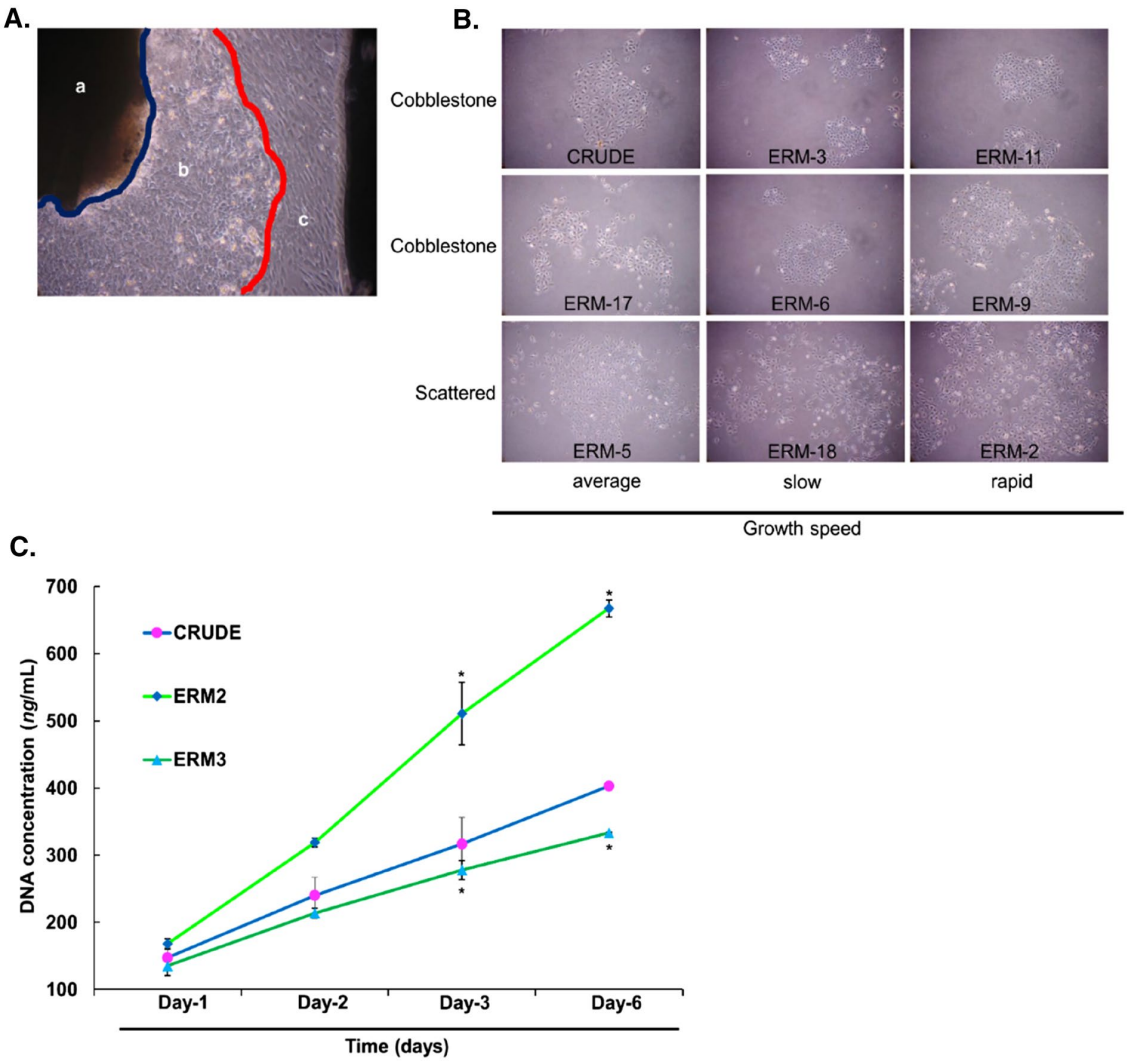
Epithelial-like cells outgrowth from the periodontal ligament (PDL) tissue explant and isolated clones from epithelial-like cells. Proliferation assay of CRUDE ERM, ERM-2, and ERM-3 cells using a CyQUANT proliferation kit. (**A**) The image shows PDL outgrowth explant with cells growing out from the tissue at day 15; (a) PDL tissue, (b) epithelial-like cells, (c) fibroblast-like cells. PDL tissue and fibroblast-like cells were scraped, followed by washing with PBS. The epithelial-like cells were allowed to grow until confluency (magnification, 40 ×). (**B**) Eighteen clones were isolated from the epithelial-like cells collected from the PDL tissue. Some of the clones are shown here and categorized based on the speed (average, slow, and rapid) and pattern (cobblestone and scattered) of growth of the cells (magnification, 16 ×). (**C**) Cells were plated (10^4^ cells/well) and cultured for days 1, -2, -3, and -6. On day 3, ERM-2 showed a significantly high (*p* = 0.005) proliferation ratio compared to ERM-3. On day 6, ERM-2 and ERM-3 cells had higher (*p* = 0.032) and lower (*p* = 0.024) proliferation ratios, respectively. (n = 3).

ERM are the only epithelial-like cells that are present in the PDL space. Therefore, we isolated these cells from the PDL tissue using the outgrowth expansion method (Fig. 1A). A total of eighteen clones from the epithelial-like cells were obtained successfully using the single-cell limiting dilution method (Fig. 1B). The clones were named ERM 1–18, and the source material of the clones was named CRUDE ERM. All the clones and the CRUDE ERM exhibited epithelial-like morphologies in the primary culture. Some of the eighteen clones are shown in Fig. 1B. Variations in the attachment and growth rate of the cells were observed under a light microscope. The cells were characterized based on their attachment to the surface of the dish and the speed of cell growth (see Supplementary Table 1 online). The cell attachment was categorized as cobblestone or scattered, and the growth rate was marked as slow, rapid, or average. Working with all eighteen clones was difficult, and not all cells had unique phenotypes; hence, clones ERM-2 and -3 along with the CRUDE ERM were selected for further experiments. The growth speeds of the clones were compared with those of the CRUDE ERMs, which were considered as standard or average. The selection of the cells was performed using the following criteria: CRUDE ERM as the original cell mass from which all clones were obtained; ERM-2, the most rapidly growing cells with a scattered proliferation pattern; and ERM-3, the slowest growing cells with a cobblestone-like proliferation pattern and were most similar to CRUDE ERM.

CyQUANT proliferation assays were performed to further confirm the differences in the proliferation ratios among the three types of cells selected. On day 3, the growth rate of ERM-2 was significantly higher (*p* = 0.005) than that of ERM-3. On day 6, the ERM-2 and ERM-3 cells presented with the highest (*p* = 0.032) and lowest (*p* = 0.024) rates of proliferation, respectively (Fig. 1C).

### Cell characterization of ERM

**Figure 2:**
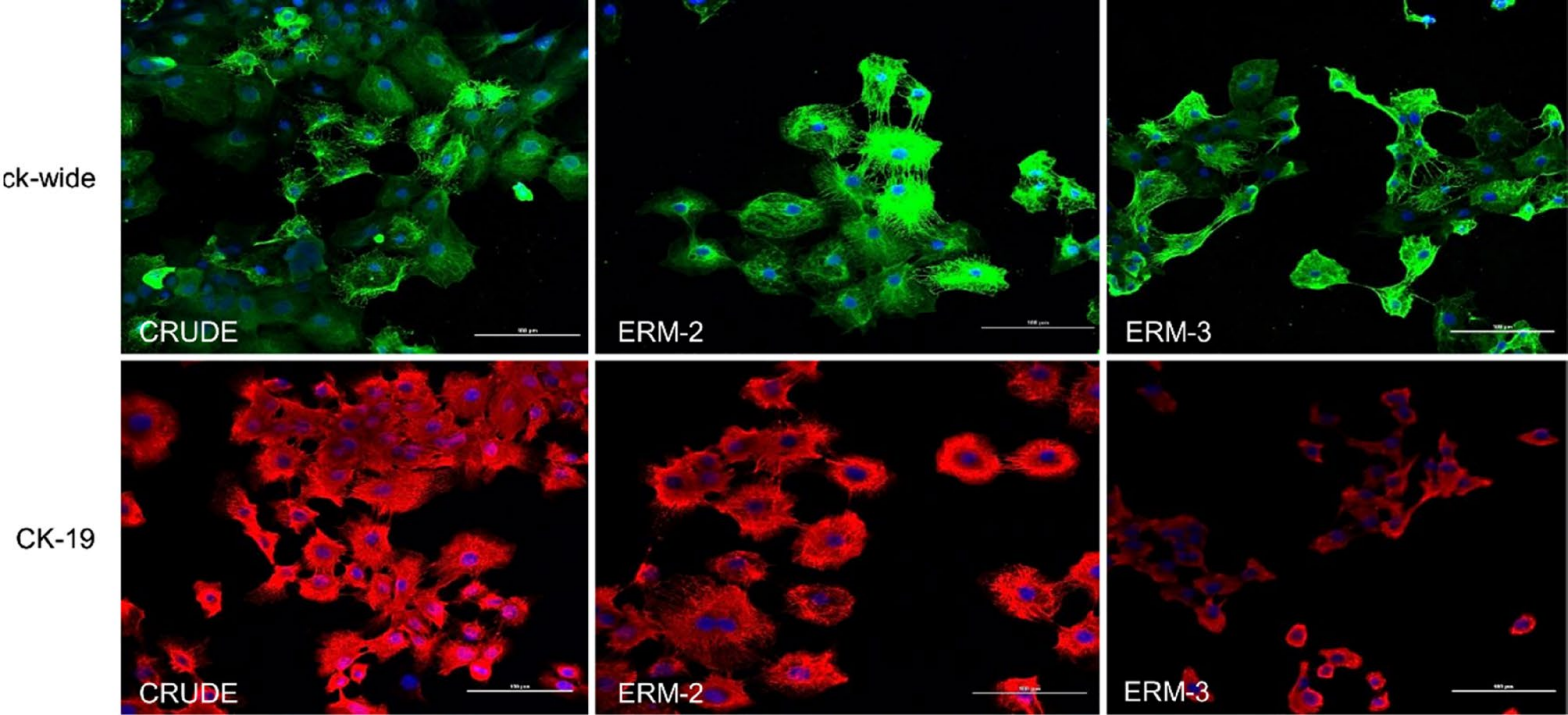
Immunofluorescence staining for cytokeratin-wide (ck-wide) and cytokeratin-19 (CK-19) in CRUDE ERM and clone ERMs. In the cytoplasm, all three cell types stained positive for both markers. The blue color represents DAPI, the green color represents ck-wide, and the red color represents CK-19. **(**Scale bar = 100 µm).

CRUDE ERM and the ERM-2 and -3 clones stained positive for both anti-wide spectrum cytokeratin (ck-wide), and anti-cytokeratin-19 (CK-19), confirming their origin as ERM cells. Keratin-positive fibers were observed in the cytoplasm of each cell isolated (Fig. 2).

### CRUDE and clone ERMs expressed ameloblast marker p75, amelogenin, and ameloblastin

**Figure 3:**
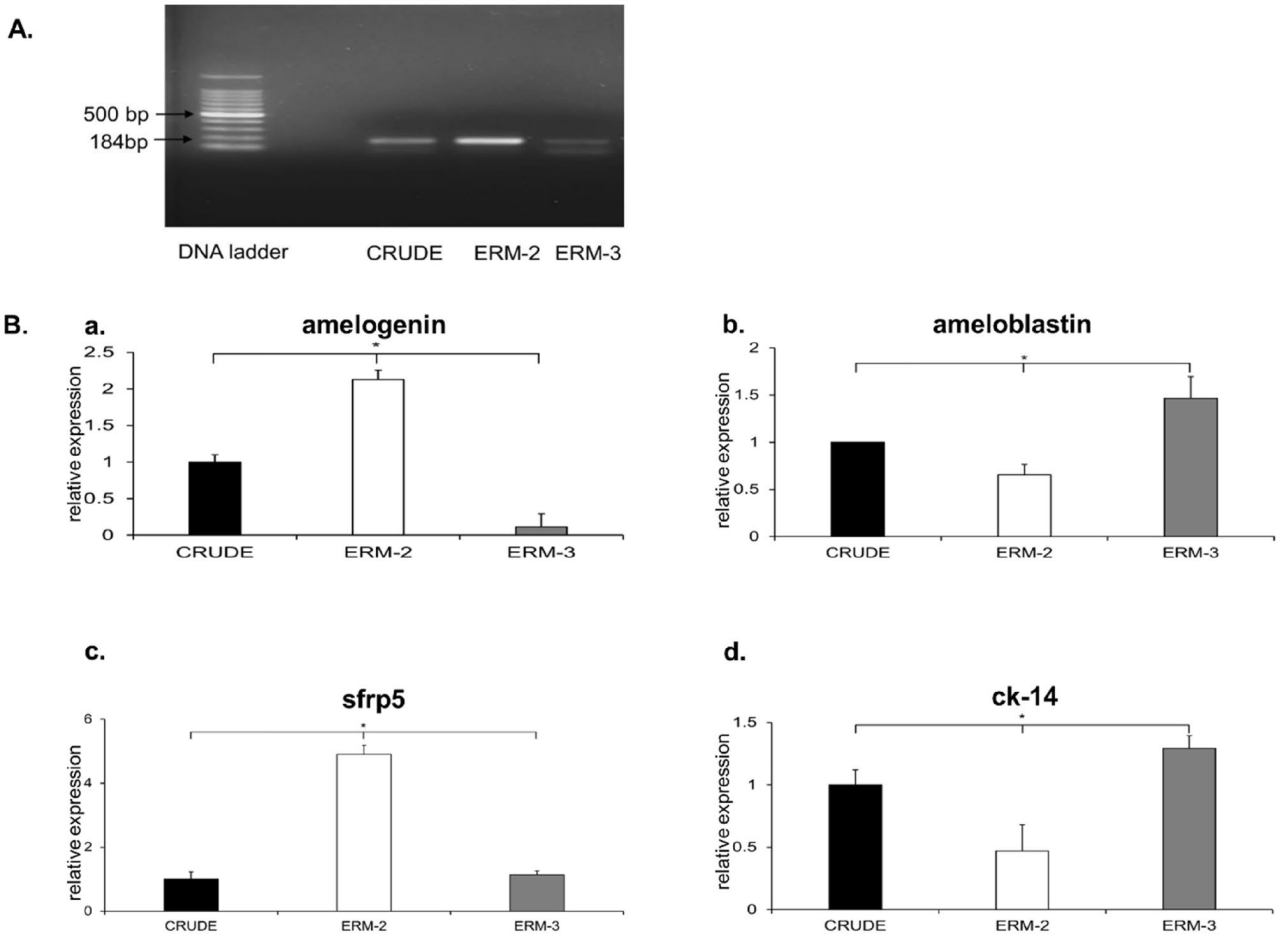
mRNA expression analysis of p75, amelogenin, ameloblastin, sfrp5, and ck-14 using RT-PCR and qRT-PCR. (**A**) A cropped image of an agarose gel demonstrating that all cells expressed for ameloblast cell marker, p75. ERM-2 and ERM-3 cells expressed at high and low levels, respectively. Full-length of the gel are presented in Supplementary Fig. 3 online. (**B**) Bar graphs comparing the levels of expression of amelogenin, ameloblastin, sfrp5, and ck-14 among the cell types. (a, b) ERM-2 exhibited the significantly highest levels of amelogenin expression compared to CRUDE ERM (*p* = 0.014) and ERM-3 (*p* = 0.013). Moreover, ERM-2 showed the significantly lowest levels of ameloblastin expression compared to CRUDE ERM (*p* = 0.025) and ERM-3 (*p* = 0.010). (c) The expression level of IEE cells marker sfrp5 was significantly higher in the ERM-2 cells compared to the CRUDE ERM (*p* = 0.025) and ERM-3 (*p* = 0.015). (d) ERM-3 showed a significantly higher level of OEE cells marker ck-14 expression when compared to the CRUDE ERM (*p* = 0.014) and ERM-2 (*p* = 0.031) cells.

Reverse transcription-polymerase chain reaction (RT-PCR) gel electrophoresis showed that the ERM-2 and -3 clones expressed the highest and lowest levels of p75, respectively (Fig. 3A). Full-length gels are presented in Supplementary Fig. 3 online.

In addition, quantitative RT-PCR (qRT-PCR) revealed that high p75-expressing cells (ERM-2) expressed high levels of amelogenin but low levels of ameloblastin compared to low p75-expressing cells (ERM-3). ERM-2 expressed the highest amounts of amelogenin, with a significant difference from CRUDE ERM (*p* = 0.014) and ERM-3 (*p* = 0.013), and the lowest amounts of ameloblastin, with a significant difference from CRUDE ERM (*p* = 0.025) and ERM-3 (*p* = 0.010), (Fig. 3Ba,b).

**Figure 4:**
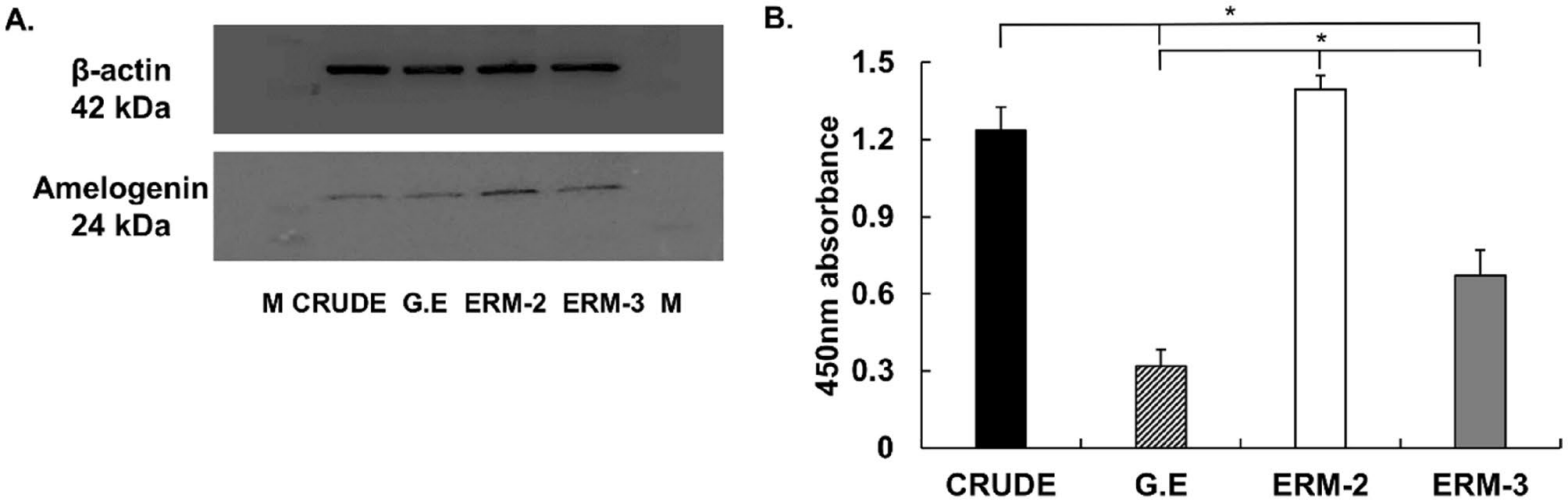
The levels of amelogenin protein production in the gingival epithelial (G.E), CRUDE ERM, ERM-2 and -3 clone cells were analyzed by western blot and ELISA technique. (**A**) Western blot analysis revealed ERM-2 cells presented with the highest expression levels of amelogenin protein when compared to the other cells. G.E cells were used as negative control and expressed the least amount of the protein among the various cell types examined. Full-length of the blot and quantification data are presented in Supplementary Fig. 4 online. (**B**) The quantity of extracellular amelogenin protein synthesis in G.E, CRUDE ERM, ERM-2, and -3 cell supernatants was determined using the ELISA method. G.E cells was used as a negative control. ERM-2 cells supernatant had the highest amounts of amelogenin protein compared to the other cells, with a significant difference from G.E (*p* = 0.008) and ERM-3 (*p* = 0.040). The amount of amelogenin protein in the CRUDE ERM supernatant differed significantly from the negative control G.E (*p* = 0.017) and ERM-3 (*p* = 0.009).

The expression profile of amelogenin protein production by western blot showed that gingival epithelial (G.E) and ERM-3 produced lower levels of amelogenin compared to the CRUDE ERM and ERM-2 cells. ERM-2 cells produced the highest amount of amelogenin among all the cell types (Fig. 4A). For a quantification diagram and full-length (polyvinylidene difluoride membrane) PVDF membrane of western blot expression, see Supplementary Fig. 4 online.

The amount of extracellular amelogenin protein expressed by cells in their supernatants was measured using the sandwich enzyme-linked immunosorbent assay (ELISA) technique. ERM-2 was shown to have much more amelogenin protein in the supernatant than G.E, CRUDE, and ERM-3, with a significant difference from G.E (*p* = 0.008) and ERM-3 (*p* = 0.040). The amount of amelogenin protein in the CRUDE ERM supernatant differed significantly from the negative control G.E (*p* = 0.017) and ERM-3 (*p* = 0.009), (Fig. 4B).

### Expression of OEE and IEE cell markers by real-time RT-PCR

The mRNA expression levels of IEE cells marker sfrp5 were significantly higher in the ERM-2 cells than CRUDE ERM (*p* = 0.025) and ERM-3 (*p* = 0.015) (Fig. 3Bc). Alternatively, the mRNA expression levels of OEE cells marker ck-14 were significantly higher in ERM-3 cells when compared to CRUDE ERM (*p* = 0.014) and ERM-2 (*p* = 0.031) (Fig. 3Bd).

### Inhibition of HPDLF mineralization in vitro

**Figure 5:**
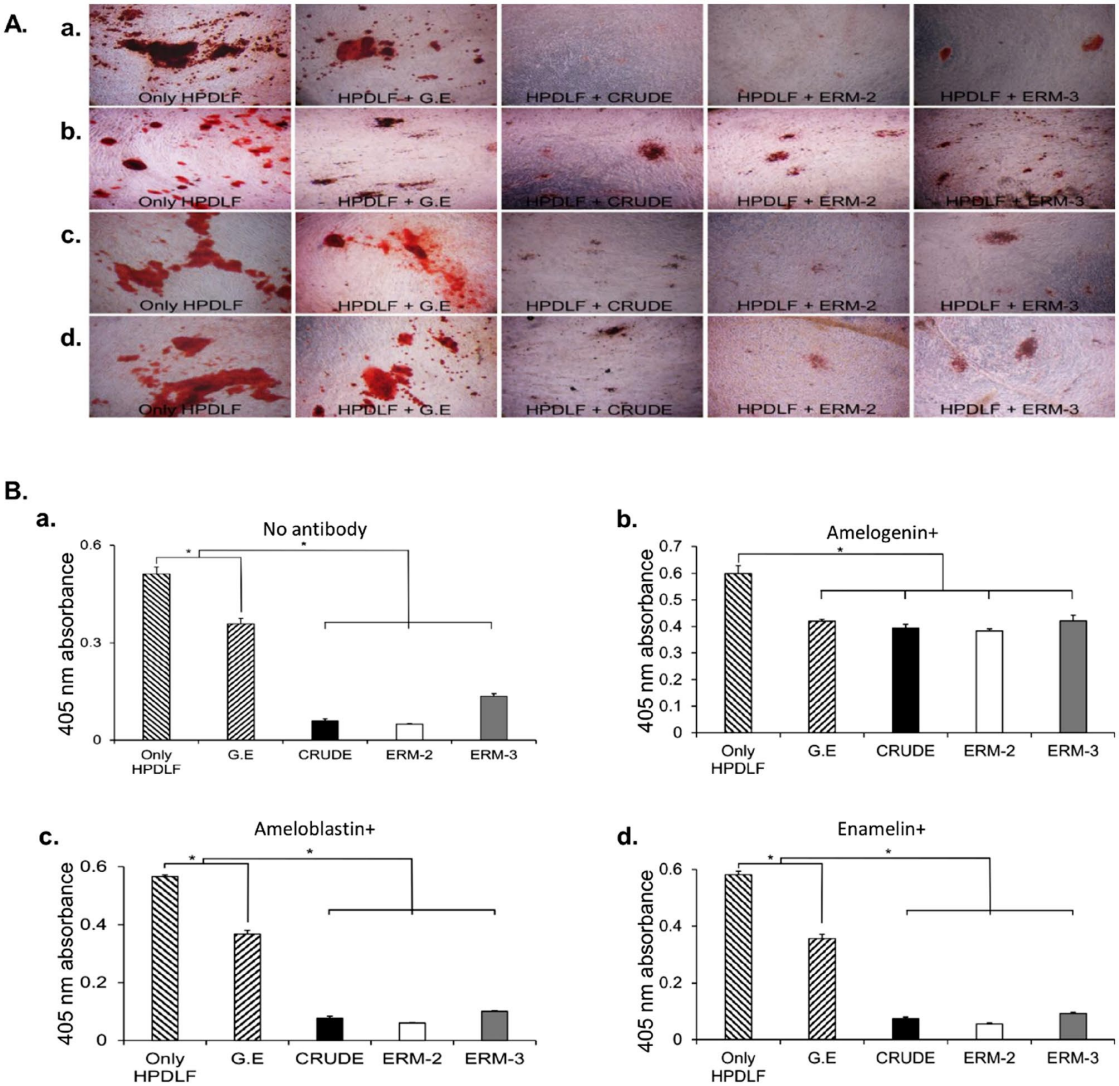
Alizarin red staining of human periodontal ligament fibroblast (HPDLF) cells co-cultured with G.E, CRUDE ERM, ERM-2, and ERM-3 cells followed by quantification of ALP activity. (**A**) Mineralization was examined by alizarin red staining of the co-culture dishes on day 30. (a) The HPDLF cells alone and those with G.E showed intense staining of alizarin red when compared to the HPDLF + CRUDE ERM and HPDLF + ERM clone cells. Mild staining was observed in the HPDLF + ERM-3 cells (b) The inhibition of HPDLF mineralization was recovered in all groups except in the control group when the anti-amelogenin antibody was added, as shown by staining with alizarin red in the cells. (c, d) The addition of anti-ameloblastin and anti-enamelin did not restore the inhibitory effects of CRUDE ERM and clone ERM cells on HPDLF cells (magnification, 16 ×). The red areas represent alkaline phosphatase staining. (**B**) Bar graphs comparing the ALP activities among the cell types. (a) Only HPDLF and G.E group showed significantly high mineralization activity compared to CRUDE ERM, ERM-2, and ERM-3 cells when no antibody was added to the medium (HPDLF: CRUDE ERM, *p* = 0.011; HPDLF: ERM-2, *p* = 0.009; HPDLF: ERM-3, *p* = 0.006; G.E: CRUDE ERM, *p* = 0.006; G.E: ERM-2, *p* = 0.044; G.E: ERM-3, *p* = 0.011). Only HPDLF group had significantly higher mineralization (*p* = 0.040) than the G.E group. (b) After the addition of the anti-amelogenin antibody, inhibition of ALP activities was restored. Only HPDLF group showed significantly higher mineralization activity compared to the G.E, CRUDE ERM, ERM-2, and ERM-3 cell groups (HPDLF: G.E, *p* = 0.022; HPDLF: CRUDE ERM, *p* = 0.006; HPDLF: ERM-2, *p* = 0.039; HPDLF: ERM-3, *p* = 0.023). (c) The addition of anti-ameloblastin failed to rescue the inhibition of mineralization. Only HPDLF and G.E groups showed significantly higher mineralization activity compared to the CRUDE ERM, ERM-2 and ERM-3 cell groups (HPDLF: CRUDE ERM, *p* = 0.016; HPDLF: ERM-2, *p* = 0.016; HPDLF: ERM-3, *p* = 0.016; G.E: CRUDE ERM, *p* = 0.042; G.E: ERM-2, *p* = 0.031; G.E: ERM-3, *p* = 0.008). Only HPDLF group had significantly higher mineralization (*p* = 0.013) than the G.E group. (d) The addition of anti-enamelin also failed to rescue the inhibition of mineralization. Only HPDLF and G.E groups showed significantly higher mineralization activity compared to the CRUDE ERM, ERM-2 and ERM-3 cell groups (HPDLF: CRUDE ERM, *p* = 0.016; HPDLF: ERM-2, *p* = 0.016; HPDLF: ERM-3, *p* = 0.017; G.E: CRUDE ERM, *p* = 0.043; G.E: ERM-2, *p* = 0.023; G.E: ERM-3, *p* = 0.008). Only HPDLF group had significantly higher mineralization (*p* = 0.039) than the G.E group.

To identify the EMPs of ERM involved in inhibiting the mineralization of HPDLF cells, anti-amelogenin, anti-ameloblastin, and anti-enamelin antibodies were added to the culture system, and the relative extent of mineralization was examined by alizarin red staining (Fig. 5A). On day 30 of the staining, HPDLF cells co-cultured with no cells and G.E cells showed intense staining, whereas those co-cultured with CRUDE or other clone cells showed no mineralization, except some staining was observed in the ERM-3 cells group (Fig. 5Aa). The inhibition of mineralization in HPDLF cells following exposure to CRUDE ERM and clone ERMs was stopped when anti-amelogenin was added to the culture media (Fig. 5Ab). However, recovery was not possible when anti-ameloblastin or anti-enamelin antibodies were added (Fig. 5Ac,d). The results of the quantification of alkaline phosphatase (ALP) activity were consistent with those of the alizarin red staining (Fig. 5B).

**Figure 6:**
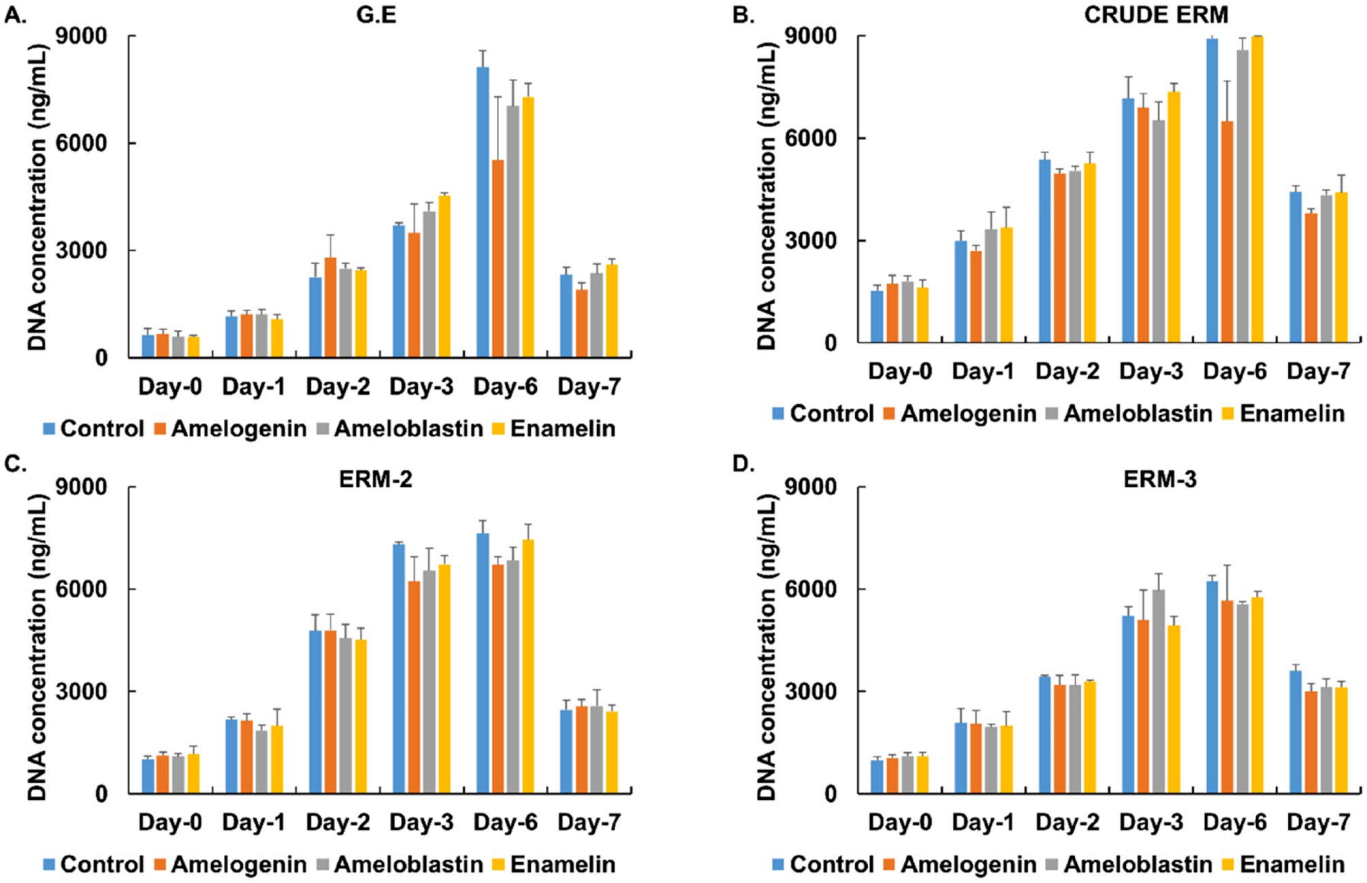
Proliferation assay of the G.E, CRUDE ERM, ERM-2, and ERM-3 cells after addition of amelogenin, ameloblastin, and enamelin antibodies (1:500, 1:2000, 1:1000) to the culture medium. Cells cultured without any antibodies in the medium were used as control. Regardless of antibody changes, there was no discernible difference in the proliferation ratio of any of the cells (**A** G.E, **B** CRUDE ERM, **C** ERM-2 and **D** ERM-3).

Proliferation assays were performed to determine whether or not the cells remained functioning after adding antibodies to the culture medium. All cells remained actively proliferative and showed no discernible variation in their proliferation ratio upon the addition of amelogenin, ameloblastin, and enamelin antibodies (Fig. 6).

### Prevention of dentoalveolar ankylosis in transplanted rat molars regulated by the level of amelogenin secretion from ERM clones

**Figure 7:**
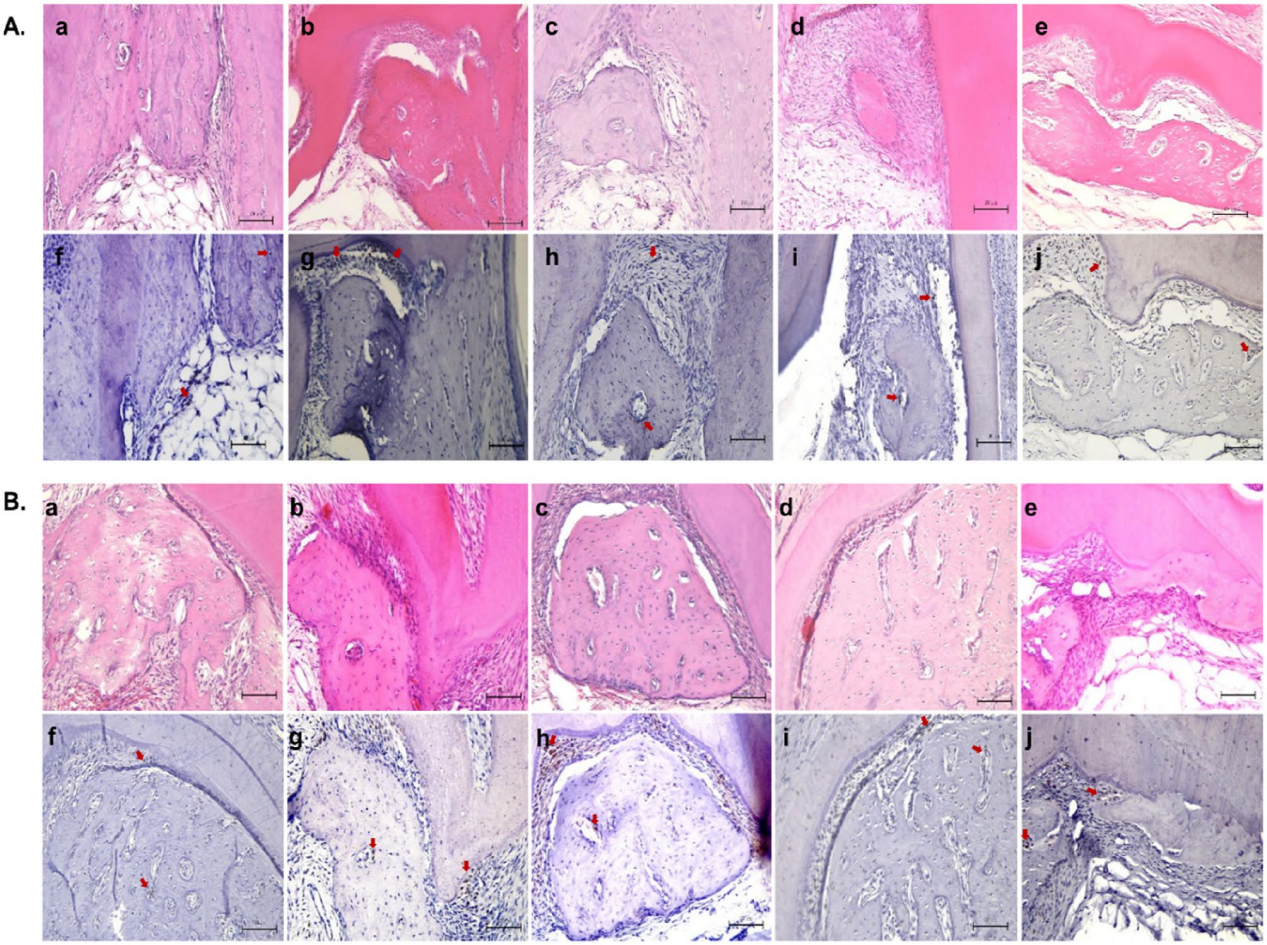
H & E and IHC staining of newly formed bone in the furcation region of a transplanted rat maxillary first molar cultured in supernatants of G.E, CRUDE ERM, ERM-2, and -3. (**A**) H & E and IHC (anti-Osterix) staining of newly formed bone in the furcation region of the maxillary first molar of the rat cultured in G.E, CRUDE ERM, ERM-2, and -3 supernatants without amelogenin antibody. H & E staining (a-e), anti-Osterix staining (f–j) were done on serial sections from the same sample. (a, b) The furcation region of the control group (fresh KBM and G.E supernatant) demonstrated larger areas of bone formation and dentoalveolar ankylosis. (c, d, e) Teeth cultured in supernatants from CRUDE ERM and ERM-2 cells formed smaller areas of new bone compared with those cultured in supernatants from ERM-3 cells; none of the experimental groups developed dentoalveolar ankylosis. In all groups (f–j), the bone neogenesis marker osterix stained positive. (Scale bar = 100 µm), (magnification, 200 ×). (**B**) H & E and IHC staining of the newly formed bone in the furcation region of the rat molars cultured in the supernatants of G.E, CRUDE ERM, ERM-2, and -3 with the amelogenin antibody (1:500). H & E staining (a–e), anti-Osterix staining (f–j) were done on serial sections from the same sample. Inhibition of new bone formation in the furcation region by amelogenin protein present in the cell supernatants was rescued by adding an anti-amelogenin antibody (a-e). All groups showed positive staining for anti-Osterix (f-j). (Scale bar = 100 µm), (magnification, 200 ×).

Four weeks after transplantation, the rats were sacrificed to observe the furcation areas in the transplanted teeth via hematoxylin and eosin (H & E) staining (Fig. 7A,Ba–e). In part A of in vivo experiment, teeth cultured in the control group, fresh KBM (keratinocyte basal medium) and G.E cell-derived supernatants presented with large new alveolar bone formation along with dentoalveolar ankylosis (Fig. 7Aa,b). Large bone formations were observed in the CRUDE ERM and ERM-3 groups (Fig. 7Ac,e), whereas the ERM-2 groups presented with comparatively small bone formations (Fig. 7Ad). No ankylosis between the newly formed alveolar bone and tooth root was observed in any of the experimental groups (Fig. 7Ac,d,e). The CRUDE ERM and ERM-2 groups, which presented with the highest secretion of amelogenin in the supernatant, formed significantly smaller bone compared to the KBM and G.E groups, and the ERM-3 groups (which had the lowest secretion of amelogenin in the supernatant). In part B, the addition of anti-amelogenin antibody eliminated the influence of amelogenin protein contained in the cell supernatant on bone inhibition. Ankylosis was detected in KBM, G.E, and ERM-3 cells supernatant cultured teeth, and large bones were formed in all groups (Fig. 7Ba-e). The number of samples showing ankylosis and bone formation in the furcation area is illustrated in Supplementary Table 3 online. Immunohistochemical (IHC) staining with osterix antibody was done as a marker for new bone formation (Fig. 7A,Bf–j). Quantification of newly formed bone during in vivo transplantation of rat molars with or without the addition of anti-amelogenin in the cell supernatants was performed and is shown in Supplementary Fig. 5 online.

### Identification of differentially expressed genes from NGS

Gene set enrichment analysis (GSEA) is a method for discovering groups of genes that are upregulated or downregulated in a large collection of genes and may be related to each other. NGS datasets from CRUDE ERM, ERM-2, and -3 were used for the analysis. Log2 FC (fold change) ≥ 1.5 or ≥ − 1.5 was used as a cut-off parameter for the detection of differentially expressed genes (DEGs). The data were divided into three groups as follows: ERM-2 vs. CRUDE ERM (a), ERM-3 vs. CRUDE ERM (b), and ERM-2 vs. ERM-3 (c). ERM-2 and -3 compared to CRUDE ERM and ERM-2 compared to ERM-3 showed 136, 900, and 841 DEGs, respectively. ERM-2 and ERM-3, compared to CRUDE ERM, had 67 DEGs in common. Moreover, these 67 common genes in between (a + b) were compared with group c and revealed 15 DEGs in common. The overview of the identification of 15 commonly shared DEGs are shown in Supplementary Fig. 6 online using the Venn diagram. The expression level of these 15 genes in CRUDE, ERM-2, and -3 was analyzed and plotted using a heat mapper (see Supplementary Fig. 7 online).

### The protein–protein interaction (PPI) network construction and identification of hub nodes

**Figure 8:**
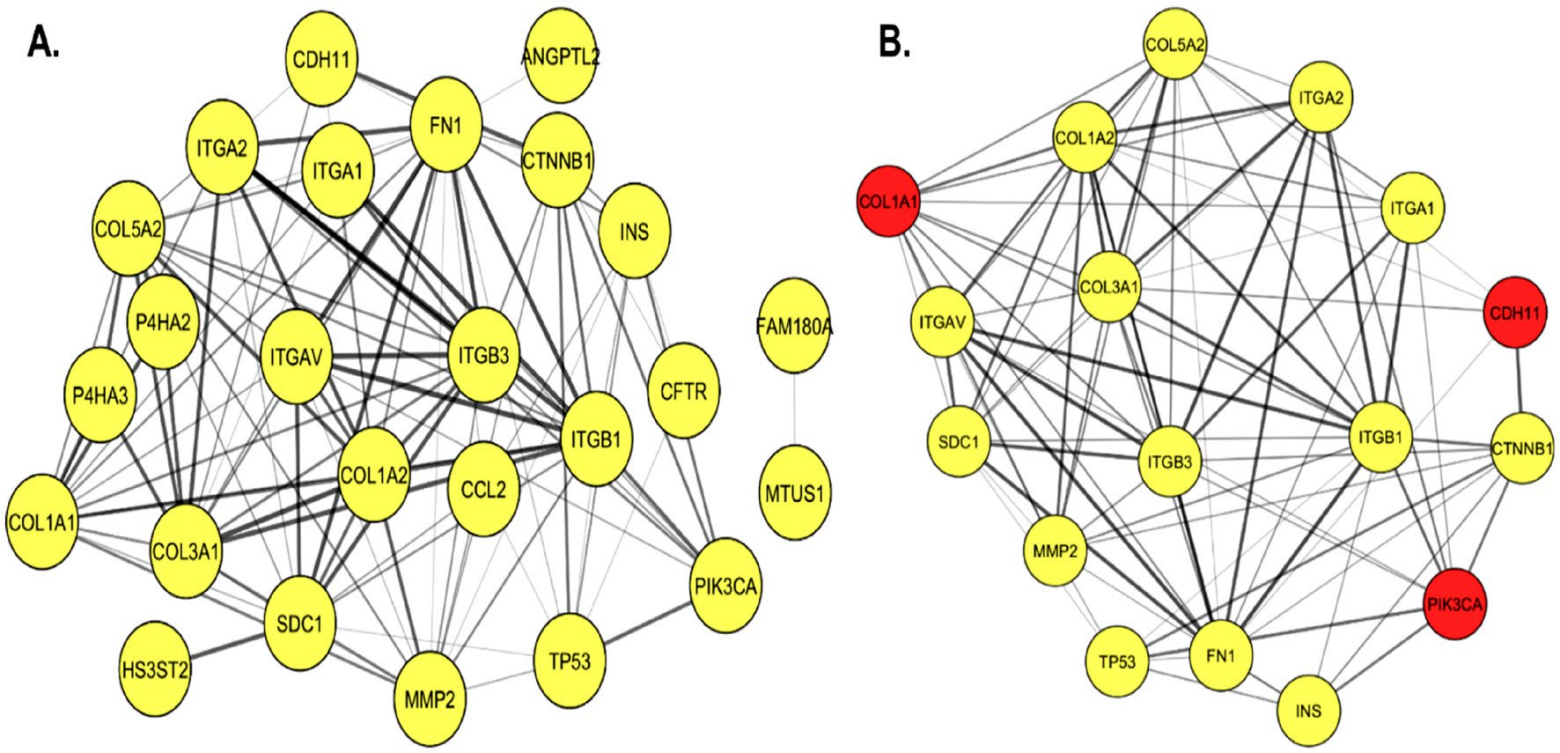
Protein–protein interaction (PPI) network construction of identified DEGs using Cytoscape and the STRING database. The nodes represent genes, while the lines here represent the interaction between nodes. (**A**) PPI network made up of 15 discovered DEGs. It was made up of 26 nodes and 103 edges. (**B**) Significant modules from the PPI network accessed via the MCODE plug-in. The MCODE modules included 17 nodes and 86 edges. This cluster shows three hub or key genes, COL1A1, CDH11, and PIK3CA, highlighted in red. The MCODE plug-in scores are shown in Supplementary Table 5 online.

PPI network of the DEGs was constructed using Cytoscape software and the STRING database. The PPI network of DEGs consisted of 26 nodes and 103 edges (Fig. 8A). The Cytoscape tool MCODE (Molecular Complex Detection) was used to screen hub genes in the network, with a cluster score of ≥ 10 as the inclusion criterion. The MCODE modules included 17 nodes and 86 edges (Fig. 8B). COL1A1, CDH11 and PIK3CA were found as the hub genes. The MCODE plugin scores are briefly shown in Supplementary Table 5 online.

## Discussion

To the best of our knowledge, this is the first study to isolate and characterize clones from the ERM using the single-cell limiting dilution method. Eighteen different clones were isolated from CRUDE ERM. Variations in the proliferation of the isolated clones might depend on their maturation status within the ERM. Immature cells proliferate faster than mature or differentiated cells^19^. Therefore, the fastest (ERM-2) and slowest (ERM-3) proliferative clones were selected for the in vitro and in vivo experiments in the present study. These clones stained positive for both ck-wide and CK-19; hence, the expression of the ameloblast, IEE, and OEE cell markers (p75, sfrp5, and ck14, respectively) was evaluated. p75 is a low-affinity nerve growth factor used as a marker of undifferentiated dental epithelial or ameloblast cells^20,21^. Sfrp5 is a Wnt signal modulator and has been used as a marker of IEE cells^22^. ck-14 is a member of the keratin family with a typical intermediate filament of odontogenic epithelium, has been used as a marker of OEE cells^22^. The expression levels of p75 and sfrp5 were high in ERM-2 cells, and that of ck-14 was high in ERM-3 cells. Thus, ERM-2 might be a type of early differentiated ameloblast or IEE cell, whereas ERM-3 might be a type of late differentiated ameloblast or OEE cell. The differentiation of an ameloblast is followed by the secretion of low amounts of amelogenin and high amounts of ameloblastin^21,23^. In this experiment, ERM-2 expressed high quantities of amelogenin and low levels of ameloblastin, whereas ERM-3 expressed low levels of amelogenin and high levels of ameloblastin. These expression patterns supported the cell types of ERM-2 and-3 as early and late differentiated ameloblasts, sequentially.

ERM cells have been suggested to be involved in the maintenance of the PDL space by inhibiting bone and cementum formation^2,4^. Therefore, we examined whether those ERM cells inhibited the mineralization of HPDLF cells using the co-culture system. Emdogain (EMD), derived from the developing enamel matrix of porcine, has been used clinically for periodontal tissue regeneration^24^. Since porcine proteins have homology with human proteins, human PDLF was used in this study along with porcine ERM. CRUDE ERM, ERM-2, and ERM-3 cells significantly reduced the mineralization of HPDLF cells in the co-culture system in the current investigation. These inhibitory effects were recovered when anti-amelogenin was added to the culture media, but failed when anti-ameloblastin and anti-enamelin were added. G.E, CRUDE ERM, ERM-2, and ERM-3 remained actively proliferative upon addition of amelogenin, ameloblastin, and enamelin antibodies into the culture medium (Fig. 6). Amelogenin might be strongly involved in the inhibitory effects of mineralization. Although the expression level of amelogenin was significantly higher in the ERM-2 than in the ERM-3 or CRUDE ERM cells, no significant variation at the inhibitory level was observed among the three cell types. The expression of amelogenin protein in CRUDE ERM, ERM-2, and ERM-3 cells was detected by western blot and ELISA analyses. The production of amelogenin protein by ERM-3 and CRUDE ERM cells might be sufficient to inhibit mineralization. Faint bands for amelogenin were detected in G.E in the western blot analysis. G.E was also found to inhibit the mineralization of the HPDLF cells even though the level of inhibition was not as significant as those of the other epithelial cells. The effect of amelogenin on mineralization remains controversial, probably due to the different concentrations of amelogenin. Some in vitro studies showed that amelogenin inhibited and promoted mineralization at high and low concentrations, respectively^25^. Therefore, the low levels of amelogenin produced by G.E might be sufficient to inhibit the mineralization of HPDLF cells.

The transplantation of rat molars that were cultured in ERM-2 (high amelogenin secretive clone) cell-derived supernatants into the rat abdominal wall resulted in significantly less bone formation compared to those that were cultured in ERM-3 or CRUDE ERM cell-derived supernatants. In the control group, teeth cultured in fresh KBM medium or G.E supernatants demonstrated larger bone formation along with dentoalveolar ankylosis. Alternatively, adding anti-amelogenin antibodies to the supernatant eliminated amelogenin's impact on new bone formation. When teeth grown in KBM, G.E, and ERM-3 supernatants with amelogenin antibodies were stained with anti-Osterix, revealed that they formed substantial amounts of new bone and ankylosis. Despite the fact that amelogenin production did not change significantly between CRUDE ERM and ERM-3, teeth cultured in CRUDE ERM supernatants exhibited much less bone development. One probable reason is that CRUDE ERM is a collection of heterogeneous cells, each of which expresses the same proteins to varying degrees. These results indicate that a high concentration of amelogenin might inhibit bone formation in vivo. However, anti-amelogenin did not cause any significant differences in mineralization among the ERM-2, ERM-3, and CRUDE ERM cells in the in vitro co-culture experiments. ERM cells interact with mesenchymal cells in vivo, which could affect the expression levels of some proteins in the ERM cells. The expression levels of EMPs produced by ERM cells were altered when co-cultured with mesenchymal cells; amelogenin expression was increased in ERM cells co-cultured with dental pulp cells^26^. Furthermore, the expression levels of KLK4 an enamel matrix proteinase were upregulated in ERM cells co-cultured with fibroblasts^27^. In the current study, the supernatants were collected from cultured CRUDE ERM and ERM clones. Therefore, the in vivo data might not directly reflect the results of the in vitro co-culture experiments in our study. Although there is conflicting data about amelogenin’s effect on mineralization in vitro, in vivo studies mainly show that amelogenin induces bone and cementum formation and maintains the PDL spaces^28^. EMD consists of 90% amelogenin and aid in cementum formation, periodontal ligament regeneration, and bone formation in intra-bony defects^29^. That implies contradictory data as to inducing bone formation and maintenance of PDL space by regenerating PDL soft tissues. Pathological bone formation in the PDL space area may cause ankylosis. Hence, physiological bone formation accompanied by remodelling of bone and cementum is needed to maintain the PDL space. Amelogenin has been reported to have properties other than enamel and bone formation, such as PDL regeneration, cementogenesis and inhibition of osteoclastogenesis^30^. These effects may promote physiological bone formation and inhibit pathological bone formation. Inflammatory reactions and vascularization are vital for the induction of heterotopic bone formation^31^. EMD was reported to promote healing with minimal inflammatory response; hence, amelogenin is suspected to possess anti-inflammatory properties^32^. Studies have shown that amelogenin inhibits endothelial cell proliferation and promotes the differentiation of endothelial cells^33^. Therefore, amelogenin might play a role in inhibiting heterotopic bone formation. The bones formed around the roots in the current study represent a type of heterotopic bone formation. Thus, the amelogenin produced by ERM-2 inhibited bone formation and might be involved in preventing dentoalveolar ankylosis. Further investigations are needed to back up this claim.

NGS datasets for CRUDE ERM, ERM-2, and ERM-3 were collected and divided into three groups using bioinformatics, and DEGs were identified between the groups: ERM-2 with CRUDE ERM, ERM-3 with CRUDE ERM, and ERM-2 with ERM-3. The DEGs among the three groups were determined using log2 FC (fold change) ≥ 1.5 or ≥ − 1.5 as cut-off criteria. The Venn diagram was used to determine the 15 most frequently expressed DEGs in this cohort (see Supplementary Fig. 6 online). The identified DEGs may provide useful information about the functional differences between CRUDE ERM, ERM-2, and -3. Of these fifteen DEGs, three hub genes (CDH11, COL1A1, and PIK3CA) were found from the PPI network (Fig. 8). The expression of COL1A1 and CDH11 was higher in ERM-2 than in CRUDE ERM and ERM-3. CDH11 controls cell fate by regulating stem cell lineage determination and epithelial-mesenchymal transition^34^. This might imply that ERM-2 has stem cell-like characteristics. COL1A1 is an important extracellular matrix protein abundantly produced by ameloblasts during the secretory phase and has a clinically significant impact on bone remodelling and cell proliferation^35,36^. There is a strong association between type I collagen downregulation and severe fibrosis and ankylosis^37^, supporting our findings that ERM-3 with low COL1A1 expression exhibited greater ectopic bone formation in the PDL space. PIK3CA, the third hub gene that typically induces bone formation, was overexpressed in ERM-3 than in CRUDE ERM and ERM-2. PIK3CA functions as a promoter in macrodactyly, a debilitating congenital disorder characterized by soft tissue and bone overgrowth^38^. Inhibition of PIK3CA reduces heterotopic bone formation^39^. The higher expression of PIK3CA in ERM-3 may be correlated with substantial bone growth in the current study.

Aside from the three hub genes, several additional DEGs have been found to be associated with the bone remodelling capacities of ERM-2. These include downregulation of BGN and COL6A2 and upregulation of EGFR and FLT-1 compared to CRUDE and ERM-3. BGN is an osteogenesis promoter gene, and COL6A2 is a negative regulator of osteoclastogenesis^40,41^. EGFR is an EGF receptor continually generated by ERM cells in the PDL region and aids in the maintenance of the human periodontal gap thickness between 0.20 and 0.40 mm. EGFR promotes osteoclastogenesis by activating tyrosine kinases such as FLT-1^42^. The inhibitory effect of ERM-2 on bone formation can be attributed, at least in part, to the downregulation of BGN and COL6A2 and the overexpression of EGFR and FLT-1.

In an animal model, cell sheets generated from human PDL, umbilical vein endothelium, cementoblasts, and porcine ERM cells were wrapped around dental implants. ERM-containing cell sheets were able to produce cementum on the surface of the dental implant as well as PDL-like tissue when combined with other cell sheets. However, in the absence of ERM, the PDL apparatus was incomplete. It has been shown that ERM cells are critical for maintaining the physiological PDL space^43^. Based on the findings of this study, ERM-2 may be more successful in the formation of PDL-like tissues surrounding dental implants and in the healing of PDL.

In this study, we focused on the association between dentoalveolar ankylosis and amelogenin produced by ERM cells. Nonetheless , as discussed, several other genes may also contribute to the prevention of dentoalveolar ankylosis. Despite this limitation, the present study provides a valuable experimental model for understanding the interaction between odontogenic epithelium and stromal cells that maintain the physiological functions of the tooth, as well as a potential therapeutic approach for dentoalveolar ankylosis or regenerative medicine in the future.

In conclusion, this study identified several clones of ERM, and one of them, which produced the most amelogenin, prevented excessive bone growth in the PDL space. Based on these findings, highly amelogenin secretive ERM clones with stem cell-like properties might be utilized to treat heterotopic alveolar bone formation in the PDL region, perhaps contributing to the prevention of dentoalveolar ankylosis. However, further studies are needed to validate this speculation.

## Methods

### PDL outgrowth explant

PDL outgrowth explant culture was performed to isolate epithelial-like cells as described previously^44,45^. Two 6-month-old porcine jaws were obtained from the livestock sales division (HOKUREN Federation of Agricultural Co-operatives; Hokkaido, Japan) and transported to our laboratory on ice. Four mandibular first molars were extracted from the porcine jaws and washed twice in phosphate-buffered saline (PBS) solution. Under a dissecting microscope, the PDL attached to the middle 2/3rd of the root was separated by a scalpel and transferred to Dulbecco’s modified Eagle’s medium (DMEM, Sigma-Aldrich, St Louis, MO, USA) with 10% fetal bovine serum (FBS, Gibco, Thermo Fisher Scientific; Waltham, MA, USA) and 2% penicillin/streptomycin (PEN./STREP., Merck, Darmstadt, Germany). The dissected PDL tissues were placed at the center of a 6-well culture dish (Trueline; Nippon Genetics, Tokyo, Japan) with a minimal amount of fresh DMEM. The explants were initially left undisturbed to increase the likelihood of adherence to the surface of the dish and examined after 5 days. After incubating for 5 days at 37 °C in 5% CO_2_, 500 µL of culture medium was carefully added to avoid floating the tissues on the culture dish. The medium was changed every 72 h until the PDL cells were sub-confluent; two different layers of cells were visible. A clear demarcating line with epithelial-like cells on the inside and a layer of fibroblast-like cells on the outside was observed adjacent to the PDL tissue (Fig. 1A). The PDL tissue and fibroblast-like cells were scraped out and fresh medium was added after gentle washing with PBS. This was done several times until there were no cells other than the epithelial-like cells. The cells were passaged using Trypsin–EDTA (0.25%), phenol red (Gibco, Thermo Fisher Scientific; Waltham, MA, USA) solution, and those with passage numbers less than four were used for all experiments in this study. In the final phases of the experiment, where cells were utilized in cell-based assays or supernatants were collected, 0.5% phenol red-free trypsin was used (Gibco, Thermo Fisher Scientific; Waltham, MA, USA).

### Single-cell limiting dilution

Epithelial-like cells obtained from PDL tissue were cultured in a 100-mm dish at a concentration of 10^4^ cells/mL with DMEM. The supernatant from the cells was filtered and kept at − 30 °C to be used for single-cell limiting dilution. Serial dilution was performed by seeding 10 µL of the epithelial-like cells (10^2^ cells/ml) in each well of a 96-well plate along with 190 µL of the stored supernatant. After 24 h of incubation, 100 µL of the medium was removed gently by pipetting and replaced with fresh DMEM. Every other day, wells with no colonies or multiple colonies were excluded after checking under a light microscope (CKX41, Olympus; Tokyo, Japan). Wells with single colonies were trypsinized and passaged into 60-mm dishes after they reached sub-confluency.

### Cell morphology and proliferation assay

Eighteen clones were isolated, initially, and named ERM 1–18. The original cell source from where the clones were obtained was defined as the CRUDE ERM. The isolated ERM clones and CRUDE ERM were passaged into 100-mm dishes at 10^3^ cells/ml with DMEM. The cells were monitored closely every day to assess their growth patterns and proliferation ratios. Images were taken using a digital camera for categorization (Fig. 1B; PowerShot A640, Canon; Japan). Three cells including CRUDE ERM, ERM-2 and -3 were selected for further experiments. The cell selection criteria are detailed in the result section and Supplementary Table 1 online. The CyQUANT proliferation assay kit (Invitrogen, Thermo Fisher Scientific; Waltham, MA, USA) was used to re-assess the proliferation ratios of the selected cells, CRUDE ERM, and two ERM clones. The cells (10^4^ cells/well) were plated with DMEM in 96-well black-walled clear-bottomed plates (Corning Life Sciences; Sigma-Aldrich, St Louis, MO, USA ) and incubated at 37 °C in 5% CO_2_ for 1, 2, 3, and 6 days. On day 6, all samples were thawed at room temperature, and 200 µl of CyQUANT GR dye/cell-lysis buffer was added to each well. Although the same number of cells was used in each plate, the amount of DNA in each dish may vary due to the different proliferation ratios. Therefore, DNA amounts were calculated in ng/ml in accordance with the product instructions. The fluorescence activity was measured using a fluorescence microplate reader (Infinite 200 PRO, Tecan; Life Sciences, Männedorf, Switzerland) with filters for 480 nm excitation and 520 nm emission (Fig. 1C).

### Cell characterization

Several studies have used ck-wide as a marker for epithelial cells and CK-19 as an ERM cell marker^46,47^. The cells were grown on a glass chamber slide (Nunc Lab-Teks II Chamber slide, Thermo Fisher Scientific; Waltham, MA, USA) and immunolabeling was performed as described previously^48^. Briefly, the cells were incubated with the primary antibodies, rabbit ck-wide, (1:200, Thermo Fisher Scientific; Waltham, MA, USA) and anti-human CK-19, (1:200, Dako; Agilent, Santa Clara, CA, USA), and subsequently, the secondary antibodies Alexa Fluor 488 Goat anti-rabbit and Alexa Fluor 546 Goat anti-mouse (1:1,000, Thermo Fisher Scientific; Waltham, MA, USA). The cell nuclei were stained with 1 mg/mL of DAPI-Fluoromount-G (SouthernBiotech; Birmingham, Alabama, USA) at room temperature for 10 min. After mounting the coverslips, fluorescent images were captured using confocal microscopy (Fig. 2, TI2-E, Nikon; Tokyo, Japan).

### Qualitative and quantitative reverse transcription-polymerase chain reaction analysis

Total RNA was extracted from the cells via the acid guanidine thiocyanate/phenol–chloroform method, using TRizol (Invitrogen; Life Technologies, Carlsbad, CA, USA). Two micrograms of the RNA sample were reverse-transcribed (SuperScript II Reverse Transcriptase, Invitrogen, Thermo Fisher Scientific; Waltham, MA, USA) according to the manufacturer’s instructions using oligo (dT) 12–18 primers (Invitrogen, Thermo Fisher Scientific; Waltham, MA, USA).

For the quantitative real-time reverse transcription-polymerase chain reaction (qRT-PCR), aliquots of total cDNA were amplified with amelogenin, ameloblastin, sfrp5, ck-14, and GAPDH primers using the KAPA SYBR FAST qPCR Master Mix (Kapa Biosystems; Cape Town, South Africa). GAPDH was used as an internal control. Amplifications were performed using a Nano Light Cycler system (Lightcycler-480, Roche; Basel, Switzerland) using the following program settings: 30 cycles after initial denaturation for 30 s at 94 °C, annealing for 30 s at 60 °C, and extension for 1 min at 72 °C. The relative mRNA expression level of each transcript was normalized against that of GAPDH. The relative quantities of gene-specific mRNAs were calculated using the 2^−(ΔΔCt)^ method^49^.

For qualitative RT-PCR, the amplification products for p75 were run on 1.5% agarose gel, and the gel was visualized by ethidium bromide staining. Primer sequences used in this experiment have been provided in Supplementary Table 2 online.

### Western blot analysis

Protein was extracted from gingival epithelial (G.E), CRUDE ERM, and the ERM clone cells as described previously^50^. Briefly, protein samples (20 µg) were separated by electrophoresis on SDS–polyacrylamide precast gels (AnyKD Mini-PROTEAN, Bio-Rad; Hercules, CA, USA) and transferred to a polyvinylidene difluoride membrane (Immun-Blot PVDF membrane, Bio-Rad; Hercules, CA, USA). The membrane was incubated overnight with mouse monoclonal anti-amelogenin (1:500; Amelogenin [F-11], Santa Cruz Biotechnology; Dallas, Texas, USA) in Milli-Q water containing 0.2% Tween-20 and 4% skim milk at 4 °C. After washing, the membrane was incubated with appropriate horseradish peroxidase-conjugated goat anti-mouse IgG H&L (HRP) (1: 10,000; Abcam; Cambridge, UK) for 1 h at room temperature. Labeled protein bands were detected using an enhanced chemiluminescence system (LuminoGraph III, ATTO; Tokyo, Japan).

### Quantification of amelogenin protein in supernatants using ELISA

A 96-well plate was coated with 50 μL of anti-amelogenin (Amelogenin [F-11], Santa Cruz Biotechnology; Dallas, Texas, USA) antibody in coating buffer (E101, Bethyl Laboratories; Montgomery, TX, USA) for 24 h at 4 °C. The plate was washed three times with 200 μL Tween PBS, then blocked with 200 μL 3% bovine serum albumin (BSA) and stored overnight at 4 °C. Standard curves and quantification of proteins present in the supernatants of G.E, CRUDE ERM, ERM-2, and -3 were done using a BCA kit according to the manufacturer's instructions (Pierce BCA Protein Assay Kit, Thermo Fisher Scientific; Waltham, MA, USA). Triplicate standardizations, different protein concentrations, and blank samples were tested to determine the optimal amount of supernatants. The following day, the plate was washed three times with 200 μL Tween PBS, and the supernatants of G.E, CRUDE ERM, ERM-2, and -3 were added to the plate in an amount equivalent to 800 ng of proteins and incubated at 37 °C for 90 min. After washing twice with 200 μL of Tween PBS, 100 μL of goat anti-mouse IgG H&L (HRP) (Abcam; Cambridge, UK) was applied at a dilution of 1:1000 for two hours at 37 °C. 200 μL of TMB substrate (E101, Bethyl Laboratories; Montgomery, TX, USA) was added after the last five washes with Tween PBS and incubated at room temperature. The reaction was stopped with 50 µL of stop solution (0.18 M H_2_SO_4,_ Bethyl Laboratories; Montgomery, TX, USA), and the absorbance was measured spectrophotometrically at a wavelength of 450 nm (Infinite 200 PRO, Tecan; Life Sciences, Männedorf, Switzerland).

### Co-culture

HPDLF cells were kindly provided by Prof. Toshiya Arakawa from the Biochemistry Division, Health Sciences University of Hokkaido, Japan. The calcification assay was performed using HPDLF cells co-cultured with G.E, CRUDE ERM, and clone cells as described previously^9^. HPDLF cells (500 µL of 10^5^ cells/mL) were grown in twelve-well Transwell units on collagen Type 1-C coated inserts (Cellmatrix Type IC, Nitta Gelatin; Osaka, Japan) with G.E, CRUDE ERM, ERM-2, and ERM-3 cells (1.5 ml of 10^4^ cells/mL) on the bottom; G.E or no cells grown on the bottom were used as a control group (Cell Culture Insert, Transparent PET Membrane, 12-well 0.4-mm pore size, Corning Life Sciences; NY, USA). For HPDLF cells, calcification-promoting medium α-MEM (Sigma-Aldrich; St Louis, MO, USA) supplemented with 2% l-glutamine (Gibco, Thermo Fisher Scientific; Waltham, MA, USA), 1% penicillin/streptomycin (PEN./STREP., Merck; Darmstadt, Germany), 10% FBS (Gibco, Thermo Fisher Scientific; Waltham, MA, USA), 10 mM β-glycerophosphate (Sigma-Aldrich; St louis, MO, USA), 25 mg ascorbic acid (Kanto Chemical; Tokyo, Japan) was used in the inserts and DMEM (low glucose) was used in the bottom for the G.E, CRUDE ERM, ERM-2, and ERM-3 cells. The mineralization of HPDLF cells was confirmed by alizarin red staining after 10, 20, and 30 days of culture. The results for 10 and 20 days are not shown here. Alkaline phosphatase (ALP) (ARD-SET, PG Research; Tokyo, Japan) activity was quantified in the dissolved alizarin red stains via spectrophotometrical absorption at a wavelength of 405 nm (Infinite 200 PRO, Tecan; Life Sciences, Männedorf, Switzerland). To determine whether amelogenin was involved in the HPDLF cell mineralization, antibodies for major EMPs produced by ERM cells (monoclonal anti-amelogenin [1:500], ameloblastin [1:2,000], and enamelin [1:1,000]; Santa Cruz Biotechnology; Dallas, Texas, USA) were added to the culture media of the CRUDE ERM, ERM-2 and -3, and G.E cells.

To observe if the cells remained actively proliferative following the addition of the antibodies, cells were cultured with (monoclonal anti-amelogenin [1:500], ameloblastin [1:2000], and enamelin [1:1000]; Santa Cruz Biotechnology; Dallas, Texas, USA) into the culture media for seven days. Cell cultures with no antibodies were used as a control. The proliferation assay was performed using CyQUANT proliferation assay kit (Invitrogen, Thermo Fisher Scientific; Waltham, MA, USA) as previously described.

### In vivo transplantation of rat molar

CRUDE ERM, ERM-2 and -3 clones, and G.E cells were cultured on growth factor-free KBM for 4 days. The cell supernatants were filtered and stored at − 30 °C for organ culture of extracted rat teeth.

The in vivo study was approved by the animal ethics and research committee of the Health Sciences University of Hokkaido (Approval number: 47-2017). All animal experiments were performed according to the Health Sciences University of Hokkaido Committee's strict guidelines on Intramural Animal Use. The handling of the animals in this experiment complied with the ARRIVE guidelines. Twenty 4-week-old male Wistar rats were used for four weeks of transplantation. The bilateral maxillary first molars were extracted and rinsed with DMEM (without serum) to remove blood clots after anesthesia with Butorphanol tartrate (2.5 mg/kg, Meiji Seika Kaisha; Tokyo, Japan), Hydrochloric acid medetomidine (0.15 mg/kg, Zenoaq; Fukushima, Japan), and Midazolam (2 mg/kg, Teva Takeda Pharma; Nagoya, Japan) diluted in saline. Molars with root fractures were discarded. For this experiment thirty molars were divided into two parts. In part A, fifteen molars were cultured in cell supernatants only, while in part B, fifteen others were cultured with anti-amelogenin in supernatants (1:500; Amelogenin [F-11], Santa Cruz Biotechnology; Dallas, Texas, USA). Three molars were used for each sample (CRUDE ERM, ERM-2, ERM-3, G.E, and fresh KBM). Molars were then transferred to 6-well dishes and cultured for 48 h with the supernatants of G.E, CRUDE ERM, ERM-2, and -3 clones with or without the addition of anti-amelogenin; fresh KBM or G.E cell supernatant was used as a negative control. Subsequently, molars were transplanted into the abdominal subcutaneous connective tissue of the same rats they were extracted from as previously described^51,52^. At four weeks after transplantation, the transplanted molars were excised along with portions of the surrounding tissue. They were fixed with 4% paraformaldehyde in 0.1 M PBS (pH 7.4) at 4 °C for 24 h and decalcified in 0.5 mol/L EDTA (pH 7.5) for 20 days at room temperature with mild shaking. The specimens were dehydrated in increasing concentrations of ethanol and embedded in paraffin. Serial sections (thickness, 2 µm) were obtained using a sliding microtome (Leica Microtome; Wetzlar, Germany). Some of the sections from both parts of the experiment were stained with H & E (Fujifilm; Wako Pure Chemical, Osaka, Japan). Others were used for IHC staining with anti- Osterix antibody as a marker for new bone formation^53^ (Anti-Sp7/Osterix antibody, Abcam; Cambrige, UK). Light microscopy was used to examine the bifurcation region of the roots, with images captured at a magnification of 200 ×. Bone formation in each sample was quantified using the images obtained from light microscopy with the aid of the ImageJ software (https://imagej.nih.gov/ij/).

### RNA-sequencing

CRUDE ERM, ERM-2, and -3 were subjected to next-generation sequencing (NGS) to identify the genes responsible for their distinct characteristics, such as cell proliferation and the ability to regulate bone formation. Total RNA was isolated from CRUDE ERM, ERM-2, and -3 cells via the acid guanidine thiocyanate/phenol–chloroform method, using TRizol (Invitrogen; Life Technologies, Carlsbad, CA, USA). The RNA was quantified and quality tested by spectrophotometry. All samples had 260/280 absorbance ratios between 1.8 and 2.1. The RNA samples were transferred to a company (Rhelixa; Tokyo, Japan) for cDNA synthesis and NGS using their procedures.

### Bioinformatics analysis

Genes with multiple gene probe sets were averaged, whereas gene probe sets without corresponding gene symbols were deleted. DEGs were collected from three groups: ERM-2 compared to CRUDE ERM (a), ERM-3 compared to CRUDE ERM (b), and ERM-2 compared to ERM-3 (c). Log2 FC of 1.5 or − 1.5 and adj. *p* < 0.01 were considered statistically significant. An online tool (http://www.interactivenn.net) was used to draw Venn diagrams of the DEGs^54^. In addition, heatmap analysis of the identified DEGs was visualized using the web application TBtools (https://github.comwith/CJ-Chen/TBtools/releases)^55^. To further explore the potential interplay between these DEGs, they were mapped to the STRING database (https://string-db.org; version 11.0)^56^, and only interactions that enjoyed a minimum required combined score of 0.4 were set as significant. Protein–protein interaction (PPI) networks were then visualized using Cytoscape 3.8.2 (https://cytoscape.org/), an open-source bioinformatics software platform^57^. The MCODE plugin was used to identify hub genes in the constructed network. The standard for selection was set as follows: MCODE scores ≥ 10, degree cut-off = 2, node score cut-off = 0.2, max depth = 100 and k-score = 2^58^.

### Statistical analysis

The statistical analyses were conducted using a dedicated statistical software (SPSS, V 26.0; IBM, Armonk, NY, USA). Results are expressed as mean ± standard deviation. Comparison among multiple groups was performed using a one-way analysis of variance (ANOVA) and Scheffe’s test. Exact *p* values were provided when necessary, and a *p* value < 0.05 was considered statistically significant for all experiments in this study.

## Supplementary Information

Supplementary Information.
