# Supplementary material for: Analysis of the cells isolated from epithelial cell rests of Malassez through single-cell limiting dilution
Source: Sci Rep. 2022 Jan 10;12:382. doi: 10.1038/s41598-021-04091-0 (PMC8748770; doi:10.1038/s41598-021-04091-0)
Supplement: Supplementary file 1 — Supplementary Information. [file 41598_2021_4091_MOESM1_ESM.pdf]

Analysis of the cells isolated from epithelial cell rests of Malassez through single-cell limiting dilution

Syed Taufiqul Islam[1], Yoshihito Kurashige[1], Erika Minowa[1], Koki Yoshida[2], Durga Paudel[2, 3], Osamu Uehara[4], Yunosuke Okada[1], Dembereldorj Bolortsetseg[1], Sayaka Sakakibara[1], Yoshihiro Abiko[2] & Masato Saitoh[1*]

Cell name	Growth speed	Growth pattern
CRUDE	considered as standard/average	cobblestone
ERM-1	average	scattered
ERM-2	rapid	scattered
ERM-3	slow	cobblestone
ERM-4	average	cobblestone
ERM-5	average	scattered
ERM-6	slow	cobblestone
ERM-7	average	cobblestone
ERM-8	average	cobblestone
ERM-9	rapid	cobblestone
ERM-10	average	scattered
ERM-11	rapid	cobblestone
ERM-12	rapid	cobblestone
ERM-13	average	cobblestone
ERM-14	average	cobblestone
ERM-15	average	scattered
ERM-16	average	scattered
ERM-17	average	cobblestone
ERM-18	slow	scattered

Supplementary Table 1. Comparison of CRUDE ERM and clones ERM 1-18 based on visual observations and digital images.

Gene	Forward (5'-3')	Reverse (3'-5')	Product size (bp)
GAPDH	GTCGGAGTGAACGGATTTGGC	CACCCCATTTGATGTTGGCG	250
Amelogenin	AACACCACCAGCCAAACCTC	CTTCCTCCCGCTTGGTCTTG	234
Ameloblastin	CCTTGCAGCAAGTAGAGGGG	TCAAACGGGCTATTGGAAAC	145
p75	GGGAGGTTGCAAATGTGTCT	GGGCCCAAGAAATGATTACA	184
Sfrp-5	ATGGAGCATAGTGCCGATGG	CTTCCGGTCCCCACTCTCTA	102
ck-14	CCAGTTCTCCTCTGGATCGC	TCCAGTGGGATCTGTGTCCA	192

Supplementary Table 2. List of primers used in RT-PCR and qRT-PCR.

**Supernatant used to culture tooth (n=3)**	**Bone observed (without anti-amelogenin)**	**Ankylosis formed (without anti-amelogenin)**	**Bone observed (with anti-amelogenin)**	**Ankylosis formed (with anti-amelogenin)**
Fresh KBM	3	3	3	3
Gingival epithelium (G.E)	3	2	3	2
CRUDE	3	0	3	1
ERM-2	2	0	3	0
ERM-3	3	0	3	2

Supplementary Table 3. Summary of *in vivo* findings.

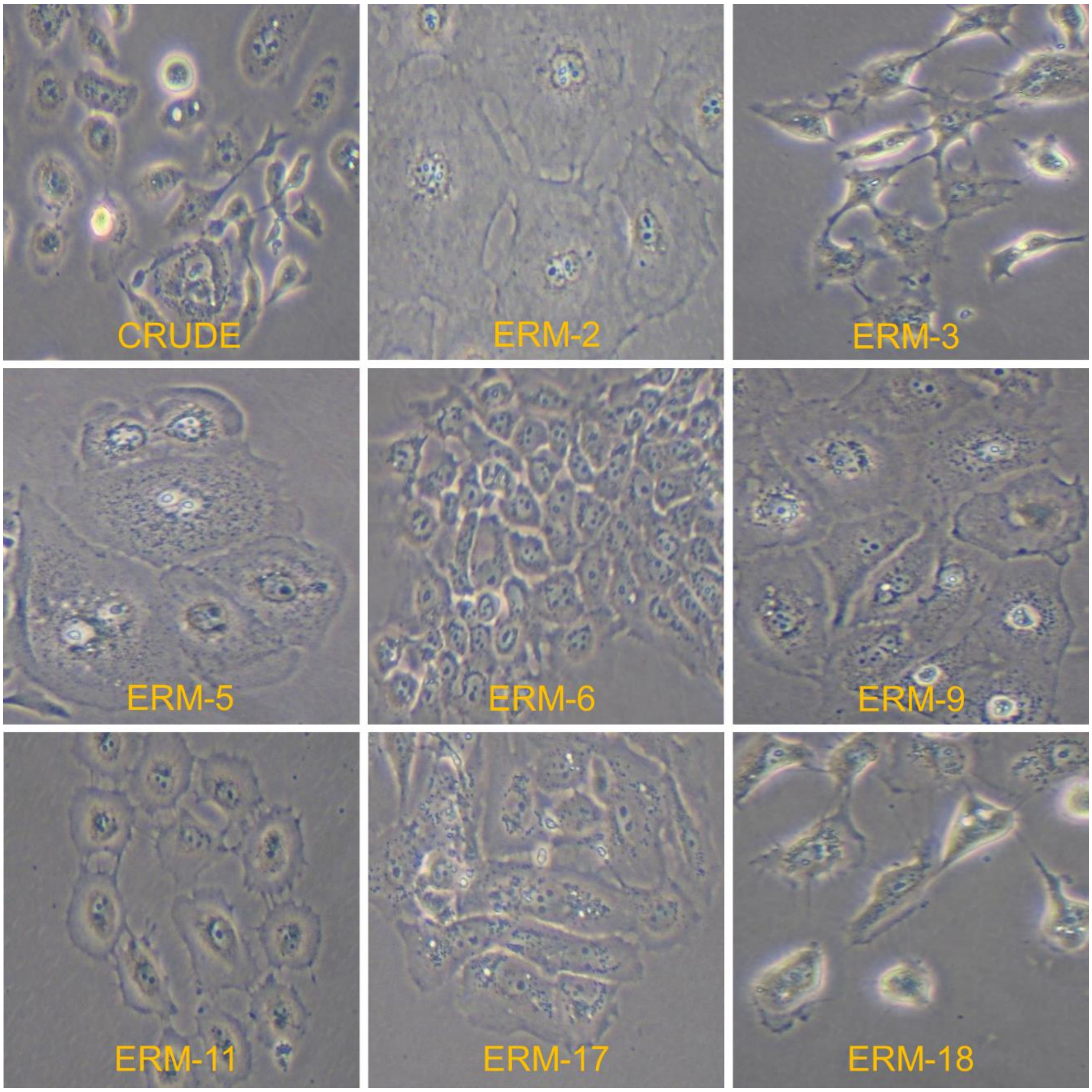

Supplementary Figure 1. High magnification images of the cells are shown in Fig.1 for morphological features. The images of the clone cells illustrate the differences in cell shape and size between them. With the exception of CRUDE ERM, all ERM clones are uniform in size and shape. CRUDE ERM shows a heterogeneous cell combination. Images were taken with a digital camera (Canon PowerShot A640; Japan) connected to a light microscope (magnification, 110x).

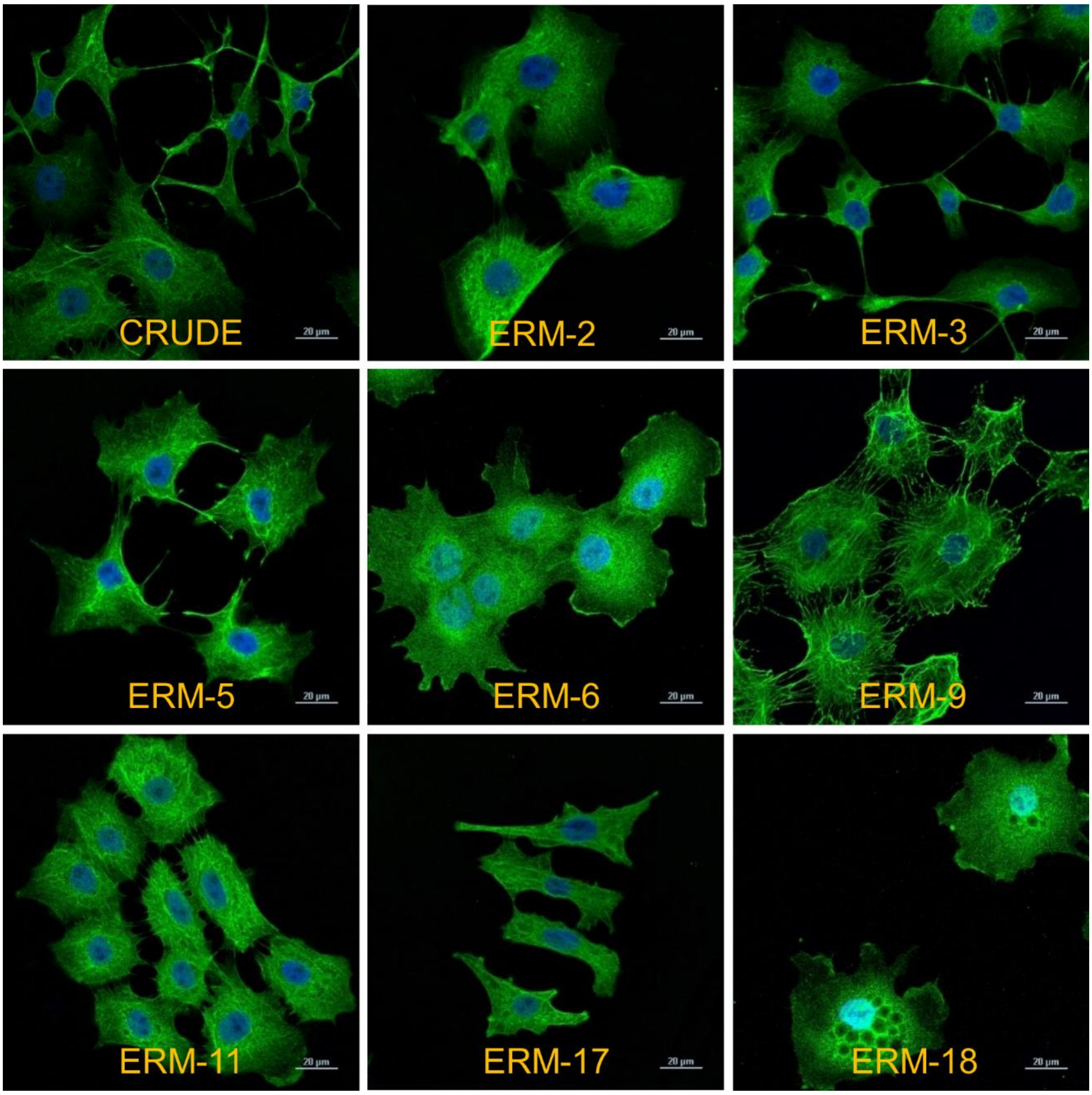

Supplementary Figure 2. Immunofluorescence images with high magnification of cells are shown in Fig.1 for morphological features. All cells stained positive for the epithelial cell marker cytokeratin-wide (ck-wide). The images of the clone cells illustrate the differences in cell shape and size between them. With the exception of CRUDE ERM, all ERM clones are uniform in size and shape. CRUDE ERM shows a heterogeneous cell combination. The blue color represents DAPI, and the green color represents ck-wide. Images were obtained by confocal microscopy (TI2-E, Nikon; Tokyo, Japan) (Scale bar=20 μm).

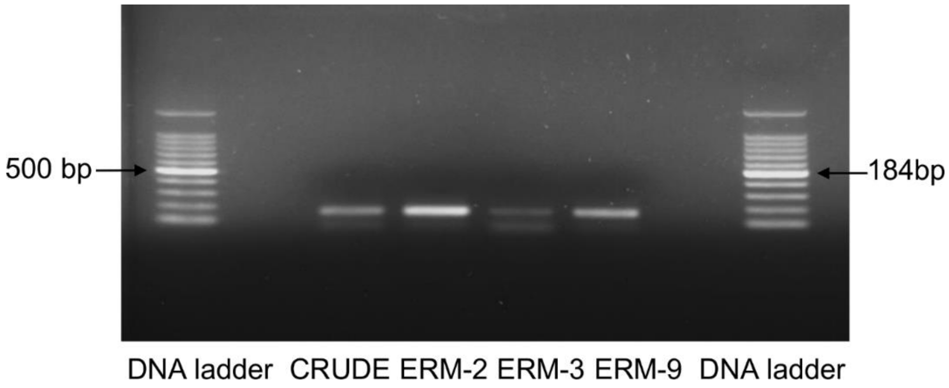

Supplementary Figure 3. Full-length gel for RT-PCR expression of the p75 mRNA. All cells expressed for p75. ERM-2 and ERM-3 cells expressed high and low levels of the marker, respectively. ERM-9 was omitted from the main text as it had no significance in the experiment.

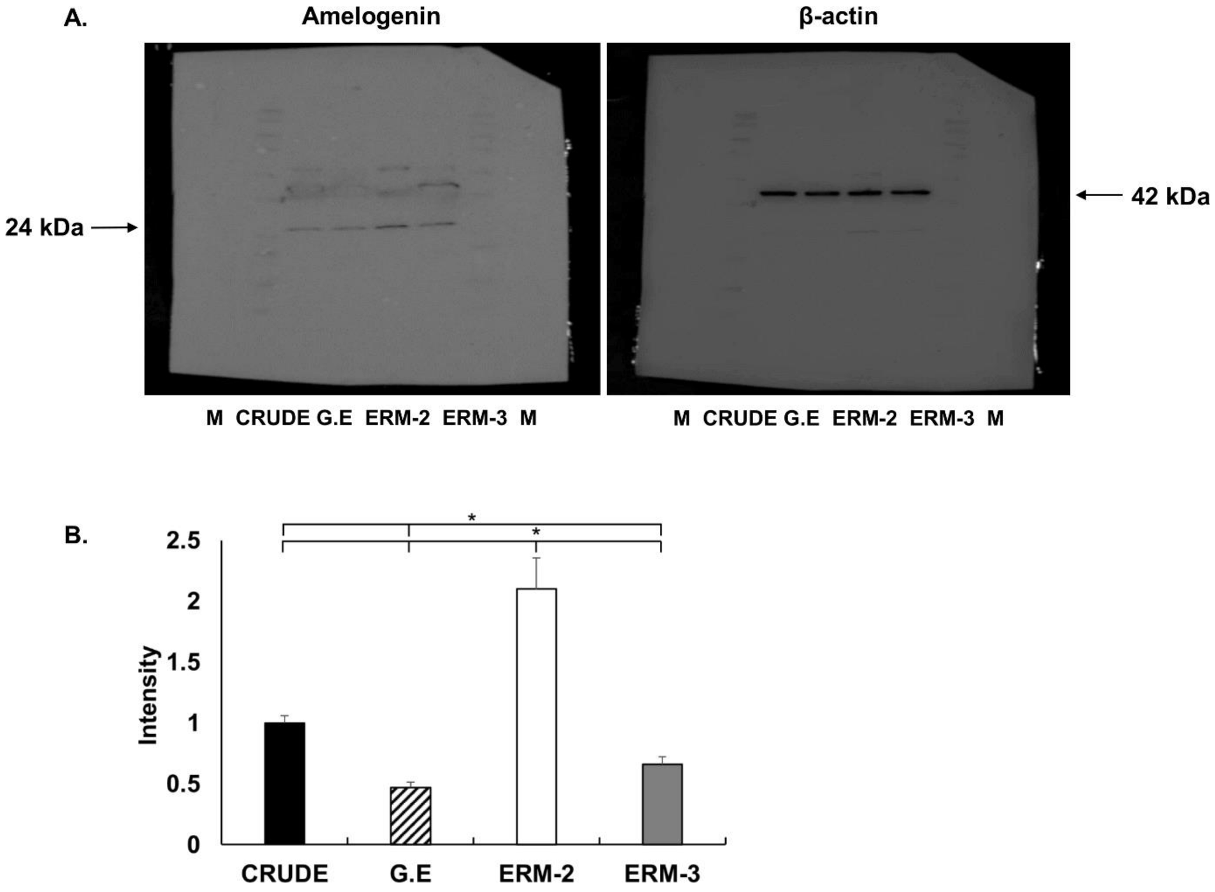

Supplementary Figure 4. A. Full-length PVDF membrane for western blot expression. After blocking, the membrane was incubated with an amelogenin antibody overnight at 4 °C with gentle shaking. Subsequently, the membrane was incubated with a secondary antibody for 1 h at room temperature. Labeled protein bands were detected using an enhanced chemiluminescence system. After three PBS washes, the same procedure was repeated with a β-actin antibody in the same membrane.

B. Amelogenin protein expression is quantified using a western blot membrane. ERM-2 produced significantly more amelogenin protein than G.E (*p*=0.028), CRUDE ERM (*p*=0.043), and ERM-3 (*p*=0.031). In contrast, CRUDE ERM produced much more amelogenin than G.E (*p*=0.023) and ERM-3 (*p*=0.008).

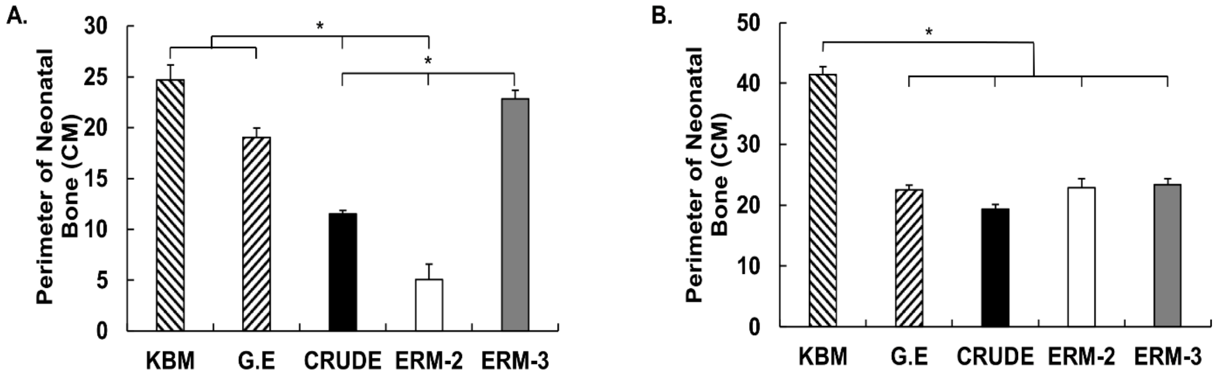

Supplementary Figure 5. Bar graph comparing the quantity of bone formation in the different groups (n = 3) with or without the addition of anti-amelogenin antibodies to the cell supernatants. The circumference of newly formed bone in the PDL region of transplanted rat molar was quantified using images (magnification, ×200) from light microscopy with the ImageJ software program (https://imagej.nih.gov/ij/). CM = centimeters. **A.** Teeth cultured without the addition of anti-amelogenin in supernatants of CRUDE ERM and ERM-2 (cells with the highest amelogenin content) demonstrated significantly lower bone formation areas than ERM-3 cells (lowest amelogenin content), G.E. cells, and fresh KBM (CRUDE ERM: KBM, p=0.042; CRUDE ERM: G.E, p=0.027; CRUDE ERM: ERM-3, p=0.036; ERM-2: KBM, p=0.044; ERM-2: G.E, p=0.033; ERM-2: ERM-3, p=0.040). **B.** In all groups, the inhibitory effect of amelogenin protein on bone formation was rescued upon adding anti-amelogenin to the supernatants, except in the fresh KBM group. Teeth cultured in fresh KBM formed significantly larger bone compared to G.E (p=0.032), CRUDE ERM (p=0.013), ERM-2 (p=0.038) and ERM-3 (p=0.010).

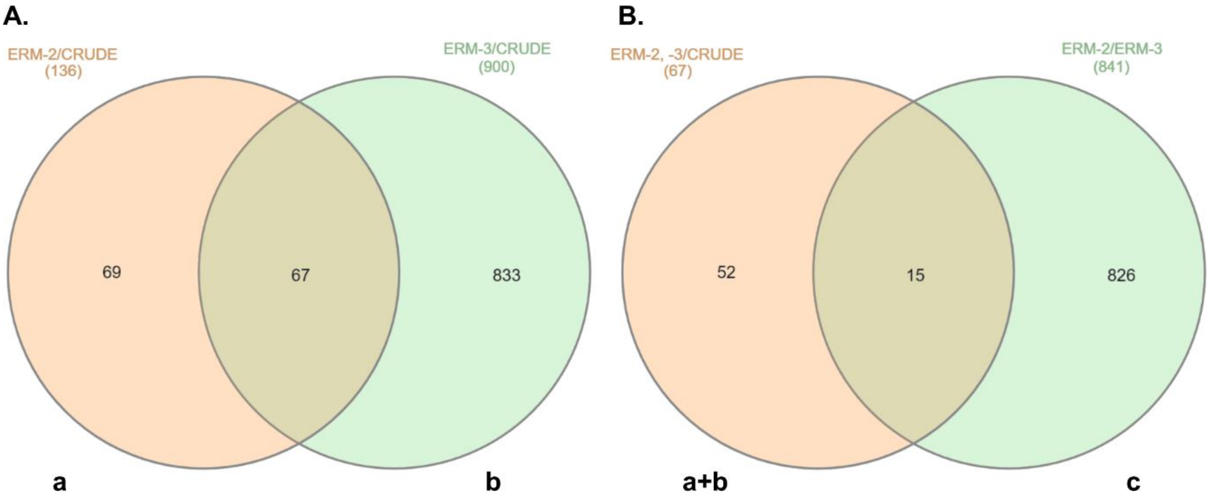

Supplementary Figure 6. A Venn diagram was used to identify common DEGs between three groups. Log2FC 1.5 or -1.5 was used as a cut-off parameter for determining DEGs. The NGS datasets were divided into three categories: a. ERM-2 vs. CRUDE ERM (136 DEGs), b. ERM-3 vs. CRUDE ERM (900 DEGs), and c. ERM-2 vs. ERM-3 (841). **A.** DEGs shared between ERM-2 and -3 with CRUDE ERM (a+b, 67 DEGs). **B.** DEGs shared by all three groups are identified. When comparing (a+b) and (c), 15 shared genes were identified in between them.

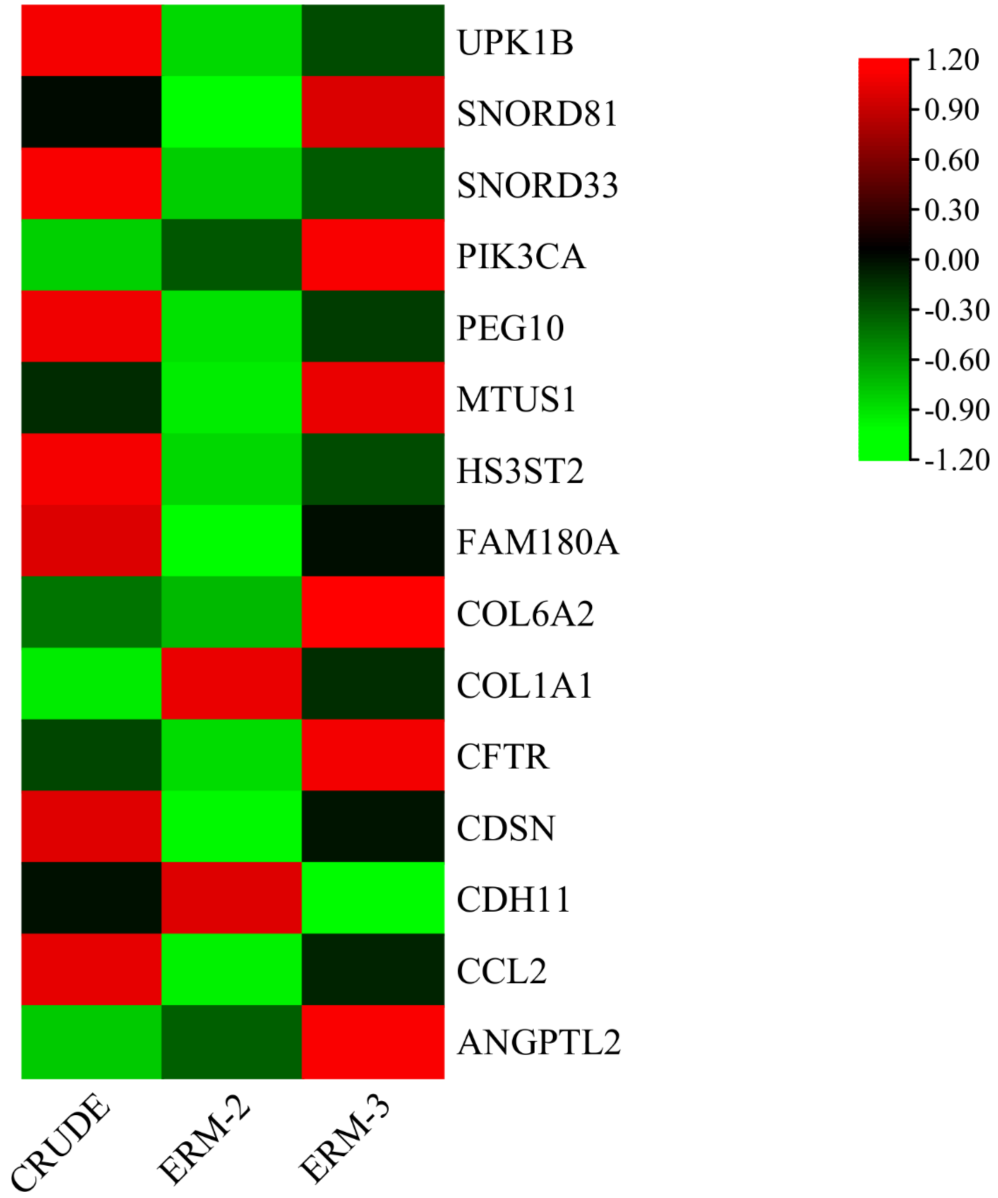

Supplementary Figure 7. Comparison of expression levels of 15 identified DEGs shared by CRUDE ERM, ERM-2, and -3 using a heatmap.

Genes	Full name	Function
ANGPTL2	Angiopoietin related protein-2	Contribute to the development of blood vessels. Over expression of ANGPTL2 causes irreversible tissue remodelling.
CCL2	C-C Motif Chemokine Ligand-2	Participate in organ regeneration and cellular calcium ion homeostasis.
CDH11	Cadherin 11	Controls stem cell differentiation and EMT.
CDSN	Corneodesmosin	Important for the integrity of the epidermal barrier.
CFTR	CF Transmembrane Conductance Regulator	Controls ion and water secretion and absorption in epithelial tissues. Promotes differentiation of osteoblasts.
COL1A1	Collagen Type I Alpha 1 Chain	Positive regulator of proliferation. During the secretory stage of ameloblast cells, it is generated in large quantities.
COL6A2	Collagen Type VI Alpha 2 Chain	Negative regulator of osteoclastogenesis. Helps to organize matrix components and is found in most connective tissues.
FAM180A	Family With Sequence Similarity 180 Member A	Phenotypes are not apparent. Found to be upregulated in fibrosarcoma and osteosarcoma.

HS3ST2	Heparan Sulfate-Glucosamine 3-Sulfotransferase 2	It is a type II integral membrane protein and possesses heparan sulfate glucosaminyl 3-O-sulfotransferase activity.
MTUS1	Microtubule Associated Scaffold Protein 1	Inhibit cell proliferation.
PEG10	Paternally Expressed 10	Negative regulator of cell apoptosis and differentiation.
PIK3CA	Phosphatidylinositol-4,5-Bisphosphate 3-Kinase Catalytic Subunit Alpha	An important part of the PI3K pathway. It's necessary for osteoblast differentiation. It's been linked to a variety of cancers.
SNORD33	Small Nucleolar RNA, C/D Box 33	It participates in molecular activities such as cytokine activity and is a structural component of the ribosome.
SNORD81	Small Nucleolar RNA SNORD81	Phenotypes are not clear.
UPK1B	Uroplakin 1B	Involved in the development, activation, growth, and motility of cells.

Supplementary Table 4. Description of commonly shared differentially expressed genes in between CRUDE ERM, ERM-2 and -3 and their functions. Three hub genes found in Fig.8B highlighted in red.

Query Term	MCODE score
Combined score= 10.62	
CDH11	4.0
COL1A1	7.56
PIK3CA	4.0

MCODE molecular complex detection, *PPI* protein–protein interaction.

Supplementary Table 5. MCODE cluster scores on PPI network of differentially expressed genes.
